# On the origin of the late-flowering *ppd-H1* allele in barley

**DOI:** 10.1007/s00122-025-04981-1

**Published:** 2025-09-10

**Authors:** Rajiv Sharma, Salar Shaaf, Kerstin Neumann, Peter Civan, Yu Guo, Martin Mascher, Michal David, Adnan Al-Yassin, Hakan Özkan, Tom Blake, Sariel Hübner, Nora P. Castañeda-Álvarez, Stefania Grando, Salvatore Ceccarelli, Michael Baum, Andreas Graner, George Coupland, Klaus Pillen, Ehud Weiss, Ian J. Mackay, Wayne Powell, Benjamin Kilian

**Affiliations:** 1 Leibniz Institute of Plant Genetics and Crop Research (IPK), 06466 Gatersleben, Germany; 2 Scotland’s Rural College (SRUC), Edinburgh, EH93JG UK; 3 University of Milan, DiSAA, Via Celoria 2, 20133 Milan, Italy; 4 UCA-INRAE UMR 1095, GDEC, Clermont Ferrand, France; 5 German Centre for Integrative Biodiversity Research (iDiv) Halle-Jena-Leipzig, Leipzig, Germany; 6 The Martin (Szusz) Department of Land of Israel Studies and Archaeology, Bar Ilan University, 5290002 Ramat-Gan, Israel; 7 The International Center for Agricultural Research in the Dry Areas–ICARDA, Beirut, Lebanon; 8 National Agricultural Research Center (NARC), Amman, Jordan; 9 Department of Field Crops, Faculty of Agriculture, University of Çukurova, 01330 Adana, Turkey; 10 Department of Plant Sciences & Plant Pathology, Montana State University, Montana, USA; 11 Galilee Research Institute (MIGAL), Tel-Hai College, 12210 Upper Galilee, Israel; 12 Global Crop Diversity Trust, 53113 Bonn, Germany; 13 Freelance consultant, 63100 Ascoli Piceno, Italy; 14 Department of Plant Developmental Biology, Max Planck Institute for Plant Breeding Research, 50829 Cologne, Germany; 15 Institute of Agricultural and Nutritional Sciences, Martin-Luther-University Halle-Wittenberg, 06120 Halle/Saale, Germany

## Abstract

To breed for climate resilient crops, an understanding of the genetic and environmental factors influencing adaptation is critical. Barley provides a model species to study adaptation to climate change. Here we present a detailed analysis of genetic variation at a major photoperiod response locus and relate this to the domestication history and dispersal of barley. The *PPD-H1* locus (a *PSEUDO-RESPONSE REGULATOR 7*) promotes flowering under long-day conditions, and a natural mutation at this locus resulted in a recessive, late-flowering *ppd-H1* allele. This mutation proved beneficial in high-latitude environments such as Northern Europe, where it allows extended vegetative growth during long spring days. We infer the origin of the mutated late-flowering *ppd-H1* allele by re-sequencing a large geo-referenced collection of 942 *Hordeum spontaneum*, 5 *Hordeum agriocrithon* and 1110 domesticated (*Hordeum vulgare*) barleys. We demonstrate that the late-flowering phenotype originated from Desert-type wild barley in the Southern Levant and present evidence suggesting a post-domestication origin of the mutated *ppd-H1* allele.

**Supplementary Information:**

The online version contains supplementary material available at 10.1007/s00122-025-04981-1.

## Introduction

Variation in days to heading (flowering time) determines the yield of crop plants by directly affecting growth stages and their development. Thus, understanding its genetic architecture is important. Barley (*Hordeum vulgare* L.) is the fourth most important cereal in terms of total production (FAO), 2024 and one of the oldest crops that was domesticated in the Fertile Crescent (Harlan and de Wet 1971; Brown et al. 1978; Zohary et al. 2015). Wild barley grains have been found in large amounts at the Ohalo II archaeological site on the shore of the Sea of Galilee and were dated to 23,000 years BP (Piperno et al. 2004; Weiss et al. 2004; Snir et al. 2015). This suggests that wild barley was collected from nature long before its domestication. Archaeological data at this site also suggest pre-domestication cultivation and provide evidence of pre-domestication cultivation and occasional failed domestication attempts (Piperno et al. 2004; Snir et al. 2015). Genetic evidence showed that the non-brittle rachis phenotype of domesticated barley originated at least three times independently (Pourkheirandish et al. 2015; Civáň and Brown 2017) and that cultivated barley has a mosaic ancestry traced to different parts of the Fertile Crescent (Pankin et al. 2014; Poets et al. 2015; Civáň et al. 2021), implying that the domestication was a protracted and spatially dispersed process. Besides the genetic analyses of extant samples, our understanding of barley domestication also benefits from genome-wide sequencing data obtained from ~ 6000-year-old seeds of domesticated barley that had been excavated from the Yoram Cave in the Masada Cliff, Israel (Mascher et al. 2016). These data provide an invaluable reference point in the study of barley domestication and diversity, as recently demonstrated by Civáň et al. (2024).

Present-day barleys are grown in diverse climatic conditions from Scandinavian countries in Northern Europe to the sub-Saharan desert in Africa and in temperate regions of the Americas and Asia (Dawson et al. 2015). The migration of the crop outside the Fertile Crescent required adaptation to these different environments (von Bothmer et al. 2003), and part of this adaptation was based on shifting the cultivation period to adapt to the optimal climatic conditions. However, the shift of the cultivation period is limited by the determinism of flowering time.

Flowering time, or days to heading (Hd) is influenced by various environmental cues, with temperature and photoperiod being the two most important in temperate cereals (McMaster and Moragues 2019). Barley has been traditionally classified as a long-day plant—that is, it flowers when daylength exceeds its critical photoperiod (typically > 14–16 h of light). However, in northern latitudes (e.g., Scandinavia, British Isles), day length > 14 h is reached in the middle of spring (April) with relatively cold temperatures, which could lead to premature initiation of flowering without a sufficient period of vegetative growth. In northern latitudes, it is therefore beneficial to delay the flowering time to summer. The species flowers in early spring in the Fertile Crescent and during long summer days in north European conditions. The transition from the vegetative to reproductive phase is controlled by a genetically diverse gene complex. Steffen et al. (2014) and Monteagudo et al. (2019) provided comprehensive reviews of key flowering genes and their pathways in cereals. Two major genes involved in photoperiod response have been identified and characterized in barley: *PHOTOPERIOD-H1*, *PPD-H1* on chromosome 2H (Turner et al. 2005) and *PHOTOPERIOD-H2*, *PPD-H2* on chromosome 1H (Kikuchi et al. 2009).

*PPD-H1* is a *PSEUDO-RESPONSE REGULATOR 7* (*PRR7*) gene that is the closest ortholog of *Arabidopsis PRR7* circadian clock gene (Turner et al. 2005) and promotes flowering time under long-day conditions. The gene was cloned by Turner et al. (2005) using a winter × spring mapping population. *PPD-H1* consists of 676 amino acids and two major conserved domains described as pseudo-receiver and a CCT (CONSTANS, CONSTANS-like and TOC1). The dominant wild-type variant *PPD-H1* accelerates flowering by upregulating the vernalization locus *VRN-H3* (*HvFT1*) (Trevaskis et al. 2006, 2007; Campoli et al. 2012). Mutation of the *Ppd-H1* allele resulted in a recessive and late-flowering *ppd-H1* variant (Takahashi et al. 1963; Turner et al. 2005). The late-flowering allele enables vegetative growth during the long days of spring and summer, which allows the cultivation period to be shifted to the warmest time of the year and thus improves yields at high latitudes.

Different terminologies have been used to describe different alleles at *PPD-H1*: (i) dominant wild-type allele (*Ppd-H1*) vs. recessive late-flowering allele (*ppd-H1*) (Turner et al. 2005), (ii) photoperiod responsive (*Ppd-H1*) vs. non-responsive (*ppd-H1*) (Maurer et al. 2015, 2016; Wiegmann et al. 2019), (iii) photoperiod sensitive (*Ppd-H1*) vs. photoperiod insensitive (*ppd-H1*) (Bustos-Korts et al. 2019), (iv) wild-type (*Ppd-H1*) vs. mutated (*Ppd-H1*) (Gol et al. 2017, 2021), and (v) wild-type (*Ppd-H1*) vs. hypomorphic (Faure et al. 2012). We follow the terminology used by Turner et al. (2005).

Two diagnostic single-nucleotide polymorphisms (SNPs) differentiating between wild-type and late-flowering alleles have been published: Turner et al. (2005) provided strong evidence for a causative SNP in the CCT domain (SNP22), while Jones et al. (2008) described another potentially causative SNP in exon 6 (SNP48). Re-sequencing different sets of wild and cultivated barleys gave contradictory results. In Turner et al. (2005) and Cockram et al. (2007), the *ppd-H1* late-flowering allele was not found in wild barley. Thus, the authors concluded that a natural mutation potentially occurred post-domestication during the spread of barley cultivation in Europe. Subsequently, Jones et al. (2008) found three late-flowering haplotypes within ‘wild’ barleys from Israel and four late-flowering haplotypes within ‘wild’ barleys from Iran, concluding that the late-flowering phenotype of European landraces originated in wild barley from Iran. However, these findings were based on a relatively small sample of potentially admixed wild barleys of genebank origins (Jakob et al. 2014). These late-flowering ‘wild’ barleys were from the east of the Fertile Crescent, and earlier studies had reported that they contributed little to the present day European barleys (Kilian et al. 2006; Morrell and Clegg 2007).

In this paper, we investigate the genetic variation at *PPD-H1* and infer the origin of the late-flowering (*ppd-H1*) allele in barley. We first performed Genome-Wide Association Studies (GWAS) of days to heading (Hd) in multi-location field trials in a diverse panel of genotypes of worldwide origin. This was followed by resequencing of the same panel at *PPD-H1* covering the potentially diagnostic SNP22 and SNP48 mentioned and debated in previous publications. Subsequently, we explored allelic diversity and re-sequenced the potentially causative genomic region around SNP22 in a comprehensive geo-referenced collection of truly wild and domesticated barley. Additionally, we gave insights into the allelic status at *PPD-H1* of the ancient 6000 years old barley sample from the Yoram cave and compared relationships among haplotypes, bioclimatic and phenotypic data.

## Materials and methods

### The genome-wide association study panel (GWAS panel)

The diverse spring barley association panel consisted of 127 two-rowed and 97 six-rowed barley genotypes of world-wide origin (Haseneyer et al. 2010; Pasam et al. 2012). One-hundred-and-nine genotypes originated from Europe, 45 from West Asia and North Africa (WANA), 40 from East Asia and 30 from the Americas (Table S1). The panel has been successfully utilized in other GWAS for agronomic traits, salt tolerance, drought stress and candidate gene-based re-sequencing studies (Long et al. 2013; Stracke et al. 2009; Comadran et al. 2012; Alqudah et al. 2014; Abdel-Ghani et al. 2019).

### Multi-location field trials (GWAS panel)

Days to heading (Hd) was scored in multi-location field trials: four locations in Germany, and one each in USA, Turkey and Syria (Table S2). As the locations were diverse, we treated four German trials as a single location in the GWAS. Hd was scored as days from the date of sowing until 50 percent of the plants had reached growth stage @GS53 (Lancashire et al. 1991). Experimental design and further details are provided in Table S2 and in Supplementary Methods.

### Markers for GWAS analysis

The GWAS panel was genotyped at TraitGenetics GmbH Gatersleben, Germany, using the high-throughput 50 k iSelect SNP chip consisting of 43,461 SNPs (Bayer et al. 2017). All allele calls were manually inspected using GenomeStudio Genotyping Module v2.0.2 (Illumina, San Diego, California). SNPs with more than 10 percent of missing values and > 10% heterozygous calls were excluded. A set of 37,387 SNPs (≥ 0.05 minor allele frequency) were used for GWAS. SNPs were anchored to the barley reference genome “Morex” assembly (Mascher et al. 2017). Details on the genome-wide association and Site x SNP interactions are provided in Supplementary Methods.

### Geo-referenced diversity panel for allele mining and phylogenetic analysis

A comprehensive geo-referenced collection (diversity panel) of 2195 wild and domesticated barley genotypes, from more than one hundred countries, was established to investigate the origin of the late-flowering (*ppd-H1*) allele in barley. This collection comprises a targeted selection of genotypes described in several publications (Badr et al. 2000; Kilian et al. 2006; Morrell and Clegg 2007; Jones et al. 2008; Pasam et al. 2012; Comadran et al. 2012; Tondelli et al. 2013; Jakob et al. 2014; Pourkheirandish et al. 2015; Russell et al. 2016; Xu et al. 2018; Bustos-Korts et al. 2019), extended by newly collected wild barleys, i.e., from Israel and Turkey (Table S3). All germplasm materials were single seed descended (SSD) for at least two generations and spikes were carefully isolated. Based on morphological and taxonomical characterization under field conditions in Germany, 138 samples were not considered for allele mining (Table S4).

For re-sequencing, we considered in total 2057 genotypes comprising (i) 942 wild barleys (*Hordeum vulgare* L. ssp. *spontaneum* (C. Koch) Thell.*, H. spontaneum*) representing the immediate progenitor of domesticated barleys; (ii) 1110 domesticated genotypes (*Hordeum vulgare* L. ssp. *vulgare*) including 433 landraces from 58 countries, 673 cultivars from 54 countries and 4 others); and (iii) five feral *H. agriocrithon* (Table S3; Supplementary Methods).

### DNA amplification and highly accurate re-sequencing at *PPD-H1*

Genomic DNA was isolated from single leaves of SSD-derived genotypes with the Qiagen DNeasy Plant Mini Kit (Qiagen, Hilden, Germany), according to the manufacturer’s instructions. The GWAS panel was re-sequenced using two primer combinations. A 1367 bp fragment was considered for analysis. The Diversity panel was re-sequenced using one primer combination only. After trimming, a fragment of 898 bp was considered for multiple sequence alignments. All details are provided in Supplementary Methods.

### Sequence analysis and detailed examination of haplotype diversity at *PPD-H1*

PCR products were purified by NucleoFast 96 PCR plates (Macherey–Nagel, Germany) and were sequenced directly on both DNA strands on Applied Biosystems (Weiterstadt, Germany) ABI Prism 3730xL sequencer using BigDye terminators. DNA sequences were processed by ABI DNA Sequencing Analysis Software 5.2 and later manually edited by BioEdit 7.2.5 (Hall 1999). Multiple sequence alignments were generated using the *PPD-H1* sequence of cultivar Igri as reference (AY970701), Turner et al. (2005).

Haplotypes were defined using DnaSP v. 5.10.01 (Librado and Rozas 2009). Singleton haplotypes were confirmed by three independent amplifications and re-sequencing.

Sequence diversity statistics was calculated using DnaSP. Diversity loss for the total number of sites (Lπ_Total_) and for silent sites (Lπ_silent_) was calculated by: Lπ = 1 − (π_domesticated_/π_wild_) (Tenaillon et al. 2004).

Median-joining (MJ) networks (Bandelt et al. 1999) were constructed using DNA Alignment 1.3.3.2 and Network 5.0.0.1 (Fluxus Technology Ltd., Clare, Suffolk, UK). SplitsTree4 version 4.15.1 was used to generate a NeighborNet planar graph of haplotypes based on the uncorrected P distances (Huson and Bryant 2006).

### Geographical distribution maps

All maps were prepared using QGIS (https://qgis.org), a free and open-source geographic information system (GIS) software. All details are provided in Supplementary Methods.

### Experiments to phenotypically characterize wild-type and late-flowering genotypes

Heading date of H10-containing genotypes was evaluated under controlled long and short-day conditionsto confirm that genotype carrying haplotype H10 possess the wild-type allele at PPD-H1. The phenotypic response to daylength (in days to heading) was tested for 41 selected genotypes including (i) 29 wild-type samples including 21 samples from GWAS panel and (ii) 12 late-flowering domesticated barley. The tested panel comprised a total of 10 different haplotypes of PPD-H1 (Table S3; Supplementary Methods).

Developmental stages under inductive long-day conditions were analyzed using data published by Alqudah et al. (2014), in which the GWAS panel was phenotyped at four developmental stages under inductive long-day conditions in the greenhouse. Thermal time was measured as growing degree days (GDD) from sowing to awn-primordium, tipping, heading and anther extrusion stages. The 218 genotypes and their haplotypes considered for this study are shown in Table S3 (Supplementary Methods).

Heading date under vernalized and non-vernalized long-day field conditions was evaluated to characterize the growth habit and photoperiod responsiveness of genotypes under contrasting vernalization treatments and long-day conditions. In total, 843 wild and domesticated barley genotypes from the diversity panel were studied in the field at IPK under vernalized and non-vernalized conditions (Table S3; Supplementary Methods).

### Inferring the allelic states of *PPD-H1* and *HvCEN* in the 6000 years old ancient barley sample JK3014 from the Yoram Cave

Sequence information from ancient seed samples (Mascher et al. 2016) were retrieved from the short-read archived accession (PRJEB12197) (https://www.ebi.ac.uk/ena/data/view/PRJEB12197). After adaptor removal and merging of overlapping paired-end sequences with leeHom (Renaud et al. 2014), merged reads with a minimum length of 30 bp were mapped to the barley reference genome (version: MorexV2, (Monat et al. 2019)) using BWA-MEM. Variants (SNP and short indels) were called from uniquely mapped reads (MAPQ >  = 20) using BCFtools (Li 2011). The genotype calls were filtered using the following criteria: (i) the minimum reads depth was 1 for homozygous calls, and (ii) the minimum reads depth was 2 in both alleles for heterozygous calls. *PPD-H1* and *HvCEN* sequences (Comadran et al. 2012) were aligned to the MorexV2 assembly by BLAST (Altschul et al. 1990). Based on the alignment result, we obtained the relative position in MorexV2 for the previously reported variants of *PPD-H1* (Turner et al. 2005) and *HvCEN*, and retrieved the genotype calls at these sites from our variant calling file.

#### Analysis of environmental data

To investigate and compare the sampling sites of key materials, 19 GIS-derived historical bioclimatic variables (mean value from 1950 to 2000) with a spatial resolution of 2.5 min (5 km) were extracted from www.worldclim.org using the R package raster v.3.0–7 (Hijmans et al. 2005a, b). Data were analyzed in R (R core team 2018) (Supplementary Methods).

## Results

### Association of Ppd-H1 with heading date across multi-location trials

**Fig. 1:**
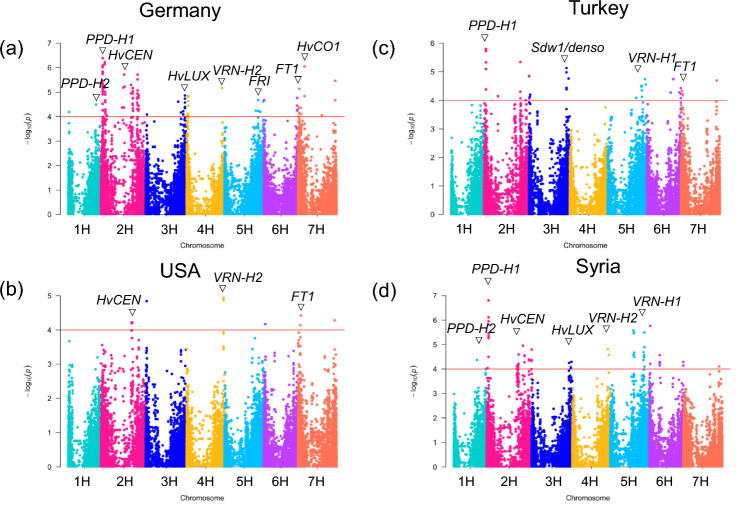
Manhattan plots of days to heading (Hd) from four trial sites are displayed. The horizontal red line shows the significance threshold based on –log_10_P = 4.0. **a** Germany; **b** USA; **c** Turkey; **d** Syria. The x-axis represents the seven barley chromosomes, while the y-axis shows the significance values of SNP associations as –log₁₀(P). Important co-localized candidate genes are indicated

Heading date (Hd) exhibited substantial variation across GWAS panel multi-location field trials, with heritability estimates ranging from 0.66 to 0.99 (Fig. S1 and Table S5). The major gene *Ppd-H1* displayed the strongest association with Hd variation, with key SNPs identified across different multi-location trials (Table S6, Fig. 1). Comprehensive details on SNP associations, allele effects, and linkage disequilibrium patterns are provided in Tables S6 and Fig. S2.

### Haplotype diversity at PPD-H1 in the GWAS panel and phenotypic characterization of haplotype H10-containing genotypes

**Fig. 2:**
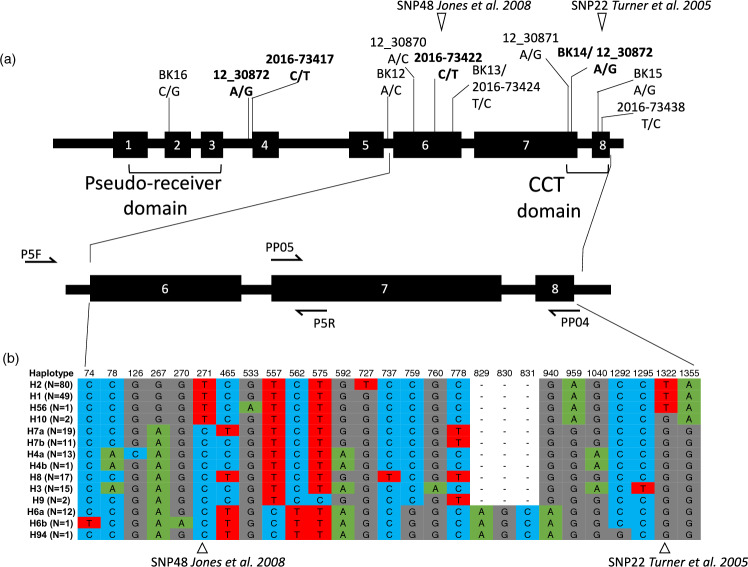
Schematic overview of *PPD-H1* gene structure and SNPs. **a** The *PPD-H1* gene consists of 8 exons. Conserved domains are indicated (Pseudo-receiver and CCT). SNP positions are shown. The 50 K SNP chip physical positions are given as in Bayer et al. (2017). Bold = significant SNPs. The locations of SNP22 and SNP48 are indicated. Primer-binding sites are shown by arrows (P5F + P5R; PP05 + PP04). **b** SNPs based on re-sequencing at *PPD-H1* in the GWAS panel and corresponding haplotypes (and their frequencies in brackets) are provided

The GWAS panel harbored 14 haplotypes (H) (Fig. 2). No strong geographical cline in haplotype frequencies was observed, except in genotypes of European origin (Fig. S8), which predominantly carried haplotypes associated with the late-flowering allele (hereafter referred to as late-flowering haplotypes; Table S7). While this pattern is most evident in northern and western Europe, late-flowering haplotypes are also present at lower frequencies in southern European accessions. Due to regional variation in sample sizes, frequencies were normalized to facilitate comparison and reduce potential bias. This pattern suggests that late-flowering haplotypes were selected in European barleys to enable vegetative growth under long-day conditions (Table S3). The most prevalent haplotypes in the GWAS panel were haplotype H2 (36%) and H1 (22%), carried by 65% and 24% of European barleys, respectively (Table S7). Barleys from the WANA region carried 11 haplotypes in total, with intermediate to low frequencies. Within American barleys, haplotypes H1 and H8 were frequent (34% and 25%, respectively), while haplotype H6b was predominant in East Asia (23%). Six haplotypes were region-specific (Table S7).

According to Turner et al. (2005) (Turner et al. 2005), plants harboring late-flowering haplotypes should carry the nucleotide ‘T’ at both positions SNP22 and SNP48, unlike the reference cultivar Igri (which carried a *PPD-H1* allele). Three haplotypes (H1, H2, H56) carried ‘T’ at both positions. However, another haplotype H10 found in two cultivars (BCC533 and BCC759, from Nepal and India, respectively) carried ‘T’ at SNP48 but ‘G’ at SNP22 (Fig. 2; Fig. S3; Table S3). To ascertain whether these two genotypes are late-flowering, they were grown together with 39 other genotypes from the Diversity panel (including 5 other genotypes carrying H10) under long- and short-day conditions (in total two haplotypes with the mutated *ppd-H1* allele and 10 haplotypes with wild-type *Ppd-H1* allele).

**Fig. 3:**
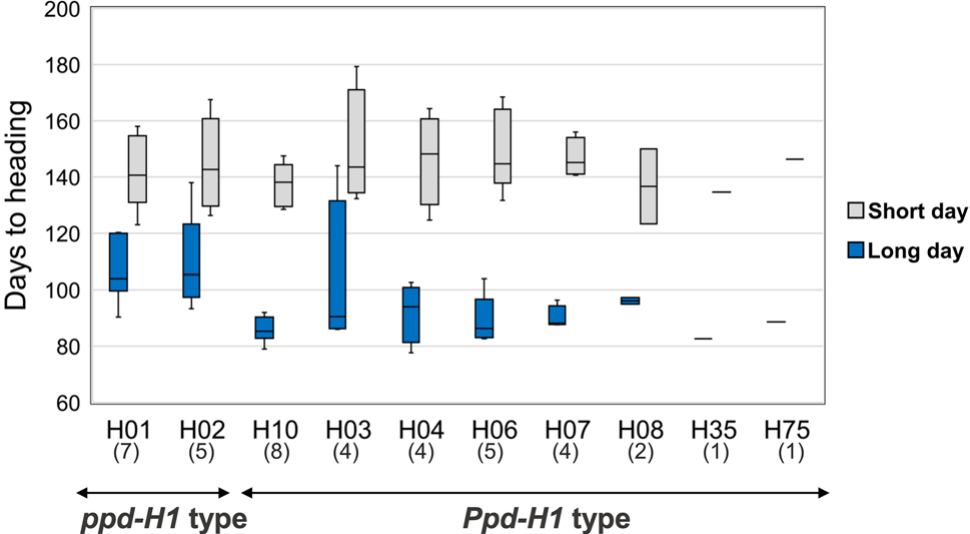
Comparison of phenotypic response (in days to heading at BBCH55) to photoperiod for 41 genotypes grown under long and short-day conditions. Differences in HD under long and short conditions are smaller for late-flowering (*ppd-H1*) genotypes compared to genotypes carrying the wild-type allele (*Ppd-H1*). Statistical significance of differences between the two allele groups was assessed using Student’s *t*-test. Boxplots are based on phenotypic BLUEs of genotypes. Accession numbers are provided in parentheses

For Hd under long- and short-day conditions, the heritability values were high with 0.98 and 0.91, respectively. While there was no significant difference among the haplotypes in short-day condition, a clear contrast was observed under long days (Fig. 3). The seven genotypes carrying haplotype H10 (Fig. 3) reached heading date significantly earlier than H1 genotypes under long-day conditions (*p* < 0.001, 22 days earlier), while under short days, the observed difference of 3.8 days was not significant. Similarly, haplotypes carrying the wild-type allele also displayed early heading under long-day conditions (Fig. 3).

Of the four genotypes carrying the haplotype H03, genotype BCC1548 exhibited a strikingly late-flowering time, showing the latest heading date under both short- and long-day conditions within the evaluated subset. Since all plants were vernalized, this delay was not due to a lack of vernalization in a facultative growth habit. According to Comadran et al. (2012), BCC1548 carries an early allele (haplotype II) at the *HvCEN* locus, which does not explain its extreme lateness. This suggests that other genetic factors contribute to the observed differences in flowering time. Nevertheless, our observations of the H10 phenology suggest that ‘T’ at SNP48 is not sufficient for late flowering under long-day conditions. Supporting our findings, the two domesticated H10-containing genotypes in the GWAS panel were identified as early-flowering under long-day conditions in the study by Alqudah et al. (2014) (Fig. S4). Accordingly, these two genotypes headed also earlier than the *ppd-H1* carrying genotypes of the GWAS panel in field trials in Germany (across four locations and two years), corroborating that haplotype H10, carrying ‘T’ at SNP48 but the wild-type ‘G’ at SNP22, indeed leads to early heading date. Therefore, our data strongly indicated that SNP22 located in the CCT domain is the causal polymorphism responsible for late flowering under long-day conditions.

### Genetic diversity at PPD-H1 within the Diversity panel

**Fig. 4:**
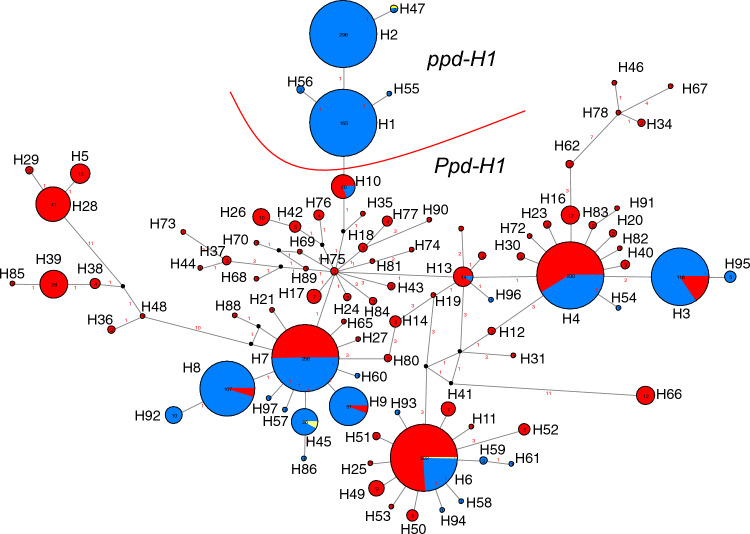
Allele mining at *PPD-H1*. Median-joining network derived from re-sequenced DNA haplotypes of 2057 geo-referenced barley genotypes. 90 haplotypes were found and are represented by arbitrarily given Arabic numerals. Circle sizes correspond to the frequency of that particular haplotype. Red, haplotype found in wild barley; blue, domesticated barley; yellow, *H. vulgare agriocrithon*. Distance in bp between haplotypes is indicated by Arabic numerals and visible at higher magnification. Eight haplotypes were shared among wild and domesticated barleys. Late-flowering (*ppd-H1*) haplotypes (H1, H2, H47, H55 and H56) cluster together. Black dots indicate median vectors. Numbers within circles correspond to the number of individuals carrying that haplotype

We detected ninety haplotypes in 2057 taxonomically confirmed SSD-derived genotypes by re-sequencing the region covering SNP22 (Figs. S5-S6; Table S8). Higher diversity was found in wild than in domesticated barleys: 71 haplotypes in wild and 27 in domesticated barleys (23 haplotypes in landraces and 17 in cultivars) (Table S8). Eight haplotypes were shared between domesticated and wild barleys. 17 haplotypes were unique to domesticated barley including the mutated late-flowering haplotypes H1, H2, H55 and H56, where *H. agriocrithon* accessions were excluded. Most haplotypes detected in wild barley had low frequencies. Only haplotypes H6 (40.44%), H4 (14.33%) and H7 (13.69%) were more frequent and were found in 68.46% of the wild barleys (Fig. 4; Table S8). These major haplotypes were not exclusive to wild barley indicating extensive post-domestication utilization (Table S3). Wild barleys from Israel contain the highest genetic diversity at *PPD-H1* (47 haplotypes), followed by Turkey (19 haplotypes). Several haplotypes were region-specific. Sixty-three haplotypes were unique to wild barley (Fig. 4; Table S3, Table S8); (Supplementary Methods). Additional figures are provided in Supplementary Methods, labeled as Supplementary Figs. S1–S15.

Within domesticated barleys, the most frequent haplotypes were H1 (15%) and H2 (27%). These two haplotypes were exclusively found in domesticated barley. The haplotype H1 and its four derived haplotypes (H55, H56, H2, H47) all carried ‘T’ at SNP22 and therefore carry the late-flowering *ppd-H1* allele. Late-flowering was confirmed by the phenotypic data from the pot experiment in the greenhouse (Fig. 3; Table S3, Table S9).

Interestingly, haplotypic diversity for wild (H = 0.79) and domesticated (H = 0.86) barleys in the Diversity panel was comparable (Table S10). Nucleotide diversity (*π*) and Waterson’s theta (*θ*) were lower for domesticated barley than for wild barley. Difference between wild and domesticated diversity was greater at silent sites (*π *silent). Within the diversity panel, 59 sites that were monomorphic in domesticated barley were found to be segregating in wild barley. By contrast, domesticated barleys carried 13 segregating sites that were monomorphic in wild material. Relative to the wild barley, domesticated genotypes display a 32% reduction of diversity at the silent sites (L*π*_silent_) and 9% reduction in the total number of segregating sites (L*π*_Total_) was observed indicating a relatively moderate diversity reduction post-domestication.

### Phylogenetic relationships and geographic distribution of haplotype H10 and derived late-flowering haplotypes

Phylogenetic relationships between 90 haplotypes were visualized using an MJ network (Fig. 4). The central position in the network is occupied by haplotype H75 carried by two wild barley genotypes from Israel (Shilat) located on the western slopes of the Judea mountain ridge. This region was previously identified as a hybrid zone between the Desert and Coast ecotypes of wild barley (Hübner et al. 2009, 2013). Radiating from here, several evolutionary trends and geographical clines were revealed (Supplementary Results File 1; Table S3).

The most interesting finding was that all late-flowering *ppd-H1* haplotypes (H1, H2, H47, H55, H56) clustered together were exclusively found in domesticated barley (and one *H. agriocrithon*) and derived from the haplotype H10 (Table S10 and Fig. 4).

**Fig. 5:**
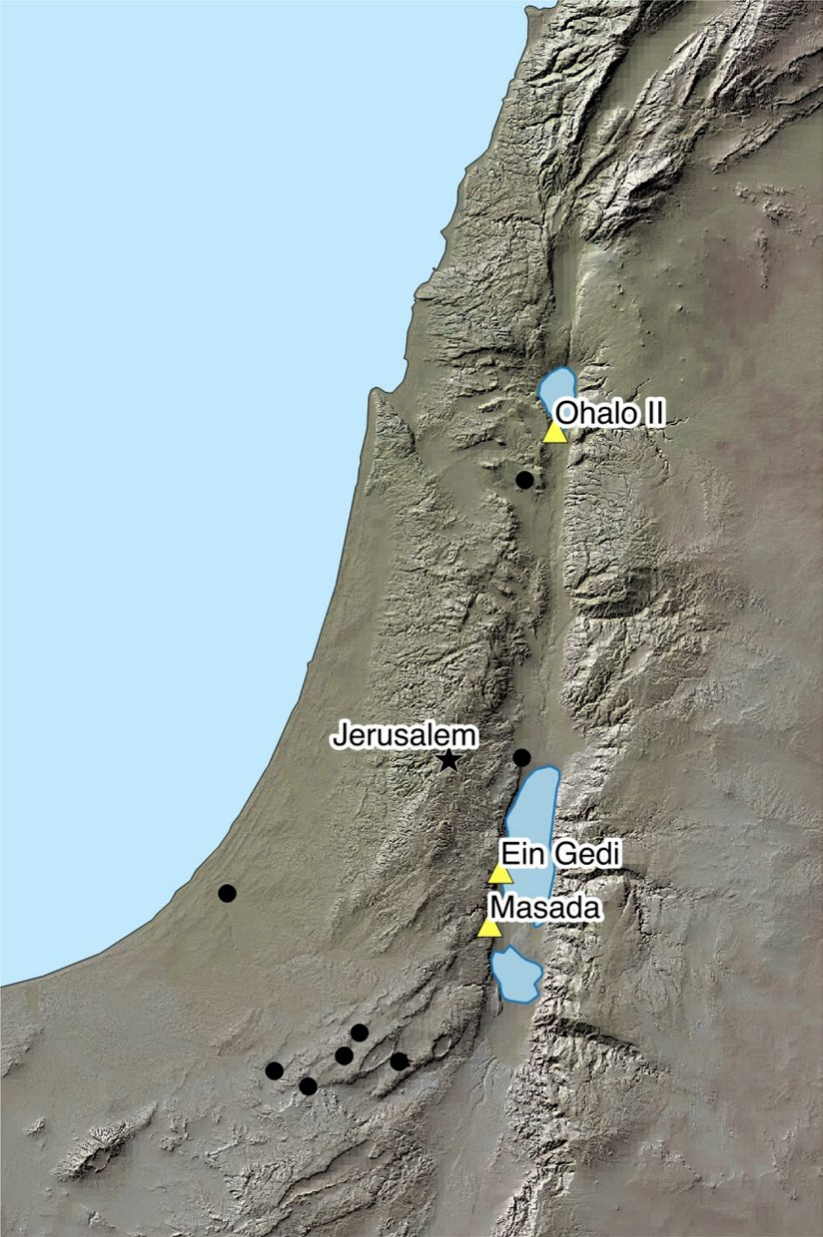
Distribution of *PPD-H1* haplotype H10 in wild barley from Israel and relevant excavation sites. Haplotype 10 collection sites are indicated by black dots. Star—location of Jerusalem; yellow triangles—excavation sites (Ohalo II, Masada, Ein Gedi)

This progenitor haplotype H10 was found only in 16 wild barleys (13 × Israel, 3 × Iran), two landraces (1 × Turkey, 1 × Nepal) and two cultivars (1 × India, 1 × Japan) (Table S3, Fig. S7). Among the wild barleys from Israel, 12 were collected in the south and east of Israel where the climate is dry and warm. They were characterized as desert ecotypes and showed early flowering (Hübner et al. 2013). One genotype was collected in the hybrid zone between the Desert and Northern ecotypes (FT138, Moledet). Most importantly, among the wild barleys from Israel were eight genotypes recently collected by Hübner et al. (2009) (Hübner et al. 2009), providing convincing evidence that haplotype H10 still exists in wild-stands in nature. The remaining four genotypes from Israel were collected in the 1960s and 1970s. Also, these showed truly wild characteristics and were collected approximately from the same locations as the eight genotypes by Hübner et al. (2009) (Hübner et al. 2009) (Fig. 5; Table S3).

Among the wild genotypes harboring the haplotype H10 were also three genotypes from Iran originating probably from two locations in the Khuzestan province. Accessions HOR2882 and IG112787 were collected by H. Kuckuck, Germany, during 1952–1954 and conserved at IPK in Gatersleben and then shared with ICARDA. One genotype (PI249983) was collected by P.F. Knowles, University of California in 1958 and maintained at USDA. Interestingly, haplotype H10 was not found in wild barley from other parts of the Fertile Crescent, including Turkey, despite extensive sampling throughout the entire distribution range of the species in Turkey (*N* = 362) (Table S3). In support of the origin hypothesis, H35, the sister haplotype of H10, was found in Israel, reinforcing the conclusion that H10 probably originated in Israel and not in Iran.

The disjunctive distribution of haplotype H10 in Israel and Iran suggests similar environmental conditions at the respective collection sites. A distinctive geographic distribution was also observed for other *Ppd-H1*-containing haplotypes, e.g., H5 was found in Israel and Iraq, or H28 was collected from Cyprus, Israel and Iran (Table S11a, S11b and S11c).

The late-flowering haplotype H1, which is evolutionary derived from H10, was found in domesticated genotypes mainly from Central Europe. Haplotype H2, which in turn is derived from H1, was mainly detected in genotypes from North-West Europe (Fig. S5 and Table S3). Interestingly, the late-flowering haplotypes H1 and H2 were found in domesticated barley from the Fertile Crescent including Bedouin landraces from Israel, newly collected by Hübner et al. (2009) (Table S3).

### The 6000 years old domesticated barley sample from the Yoram cave carried the wild-type allele at PPD-H1

We determined the allelic states at *PPD-H1* and *HvCEN* in the ancient DNA (aDNA) sample JK3014 extracted from barley grains found in the Judean desert and dated to 6000 years BP (Mascher et al. 2016). After aligning previously published aDNA sequences (sample JK3014) to the barley reference genome sequence (MorexV2, Monat et al. 2019), the putative causal variants at *PPD-H1* and *HvCEN* were determined (Table S12). The ancient barley carried the wild-type *ppd-H1*allele at *PPD-H1* and the ‘early’-flowering/proline-containing allele (C at position 531), suggesting that the Masada landraces are likely not the progenitor of the *ppd-H1* allele. A multiple sequence alignment analysis concluded that JK3014 carried the following haplotypes; haplotype H4 at *PPD-H1* and haplotype IV at *HvCEN* (Comadran et al. 2012).

### Vernalization requirement and phenotypic performance of genotypes containing haplotype H10 under long-day field conditions

The heading date of wild and domesticated genotypes was investigated under vernalized and non-vernalized conditions to determine their vernalization requirement and to characterize key agronomic traits in the vernalized treatment. For all traits, the heritability of data was high and ranged from 0.86 to 0.98.

In the non-vernalized treatment, 582 genotypes were heading, while the remaining 261 genotypes (30%) were not flowering and therefore considered winter types. On average, heading date was reached 15 days later under non-vernalized compared to vernalized conditions among the 582 genotypes. Interestingly, a bimodal distribution of Hd differences was observed, indicating two phenotypic groups: (i) spring growth habit (flowering time similar with or without vernalization) and (ii) facultative growth habit (flowering without vernalization but earlier when vernalized) (Fig. S9a). However, the potential influence of other gene interactions cannot be entirely ruled out, as the evidence we have is phenotypic rather than molecular.

Of all phenotyped 843 genotypes of barley, 97 wild genotypes did not exhibit completely wild characteristics and were excluded from subsequent analysis, leaving 746 genotypes for further comparison (470 wild and 276 domesticated). A total of 204 genotypes were classified as spring types (including 6 wild barleys from Israel) and 305 as facultative, while 237 genotypes were classified as winter types.

The genotypes carrying the late-flowering haplotypes H1 and H2 at PPD-H1 were mostly spring types. In contrast, the wild-type progenitor haplotype H10 was mainly found in facultative types (wild barley from Israel, *N* = 9) but also in two spring types (wild barley FT147 from Israel, landrace FT537 from Turkey) and one winter type (wild barley FT002 from Israel) (Fig. S9b). The genotypes harboring haplotype H10 showed a short life cycle with the second earliest heading date (1. H66, 2. H10, 3. H26) and the earliest maturity date (1. H10, 2. H66, 3. H26) under vernalized, long-day field conditions (Fig. S9c; Table S9), even when only wild barley was considered (Fig. S9d). In addition, the H10-containing genotypes were among the genotypes showing the shortest plant height, narrowest flag leaves, shortest main ear and narrowest main ear width (Table S9). From these data, we conclude that plants containing the haplotype H10 are well adapted to their local environmental conditions in the Southern Levant, and that they are characterized by facultative or even spring growth habit (Supplementary Methods).

### Analysis of environmental data sheds more light on the region of origin of the late-flowering allele

To characterize the collection sites of wild and landrace barleys, bioclimate variables were clustered by employing principal component analysis (PCA) (Figs. S10-S11 and Table S13). The precipitation-related variables, i.e., Bio14, Bio17, Bio12 and Bio18, were separated from the temperature-related variables (Bio1, Bio5, Bio6, Bio9, Bio10, Bio11) on the PC1 axis (Figs. S10-S11 and Table S13). With PC2, Bio4 and Bio7 were separated from Bio8, Bio13 and Bio16 (Fig. S10-S11). The PC1 mainly separated collection sites of late-flowering barley carrying the haplotypes H1 and H2 from wild-type allele containing barleys (Table S13). Most collection sites of late-flowering genotypes had higher values of the precipitation-related variables and lower values of the temperature variables (Fig. S12).

PCA of bioclimatic variables showed clustering of late-flowering haplotypes (H1, H2) with wetter regions, though this may reflect historical sampling locations rather than clear adaptive signals.

## Discussion

### SNP22 is the most likely causal basis of the late-flowering allele

Our GWAS results confirmed that *PPD-H1* is one of the most important genes in regulating flowering time variation as this gene causes natural diversity of barley flowering. In our study, the two functional SNPs reported by Turner et al. (2005) and Jones et al. (2008) did not show differences in terms of the significance level. This is in accordance with the observed near perfect LD between these SNPs reported by Turner et al. (2005) and Jones et al. (2008). While Jones et al. (2008) concluded that the functional SNP22 reported by Turner et al. (2005) is not causal, it should be noted that the claim was based on 87 genebank accessions only.

By re-sequencing of the gene space spanning the putative causative SNP22 and SNP48 previously reported to be in complete LD, we detected intermediate haplotypes between the two, possibly due to substitution or recombination (Fig. 2). In the diversity panel of 2057 barley samples, we found 20 (0.97%) genotypes (16 wild, 4 domesticated) showing such combination (haplotype H10), compared to Jones et al. (2008) who used a landrace set of mostly European origin and found no recombination between these SNPs. We show that H10-containing genotypes flower early under long-day conditions compared to genotypes carrying the late-flowering haplotypes (Fig. 3; Fig. S9). This shows that SNP48 by itself does not lead to late flowering, and therefore, SNP22 in the CCT domain of Turner et al. (2005) should be considered as the causal basis of the *ppd-H1* mutation. All five late-flowering haplotypes clustered together in the MJ network, thus suggesting a monophyletic origin of late-flowering in barley due to the ‘G’ to ‘T’ non-synonymous substitution at SNP22 (Fig. 4). It is important to note that in our study no phenotypically wild barley was found that carries the late-flowering allele. This is consistent with Baloch et al. (2013) who studied wild barley from Jordan and Iran. Although strong evidence suggests that SNP22 is the causal variant, further functional validation, potentially through gene editing approaches, is needed to confirm these findings.

### The late-flowering allele originated from desert ecotype of wild barley in the Southern Levant

The enormous sample size of the diversity set including a comprehensive set of newly collected wild barley enabled us to portray the geographic distribution of the variants. In total, sixteen wild barleys harboring haplotype H10 were found. They were collected from (i) Israel (*N* = 13) and (ii) Iran (*N* = 3). When we compared the environmental data for the European domesticated late-flowering barley to the niches of the H10-containing wild barley from Israel and Iran, we found that the Israeli locations are more similar to those European environments. On this basis, we conclude that H10—the ancestral haplotype to the late-flowering H1 haplotype—originated in Israel. However, we acknowledge that the observed environmental associations may be influenced by historical sampling bias and spatial clustering of accessions, potentially confounding signals of true local adaptation.

If all the above is correct, then a question arises about the occurrence of H10 in Iranian wild barley. Barley seeds, similarly, to wheat, lack features that facilitate long-distance dispersal by wind or animals (Lynch 2007). If H10 evolved in Israel, how was it then transferred to Iranian wild barley? We speculate that the province of Khuzestan in southwestern Iran was part of the ancient natural distribution range of the species and that haplotype 10 survived the Last Glacial Maximum (LGM) about 21 k years ago in the region (Jakob et al. 2014). Alternatively, another plausible explanation could be gene flow between populations over long distances along old trade routes (Fuller et al. 2012; Morrell et al. 2003; Civáň et al. 2013; Zohary et al. 2015).

For some of these H10-containing wild barleys, the haplotype of a second important flowering time gene, *HvCEN* is known form the study of Comadran et al. (2012). The following *PPD-H1*–*HvCEN* haplotype combinations were found in Israel (3 × H10–HIII, 1 × H10–HIX) and Iran (3 × H10–HI). This result supports the origin of European late-flowering barley from Desert-type wild barley from the Southern Levant, likely carrying H10–HIII, as haplotype III at *HvCEN* was by far the most frequent haplotype in European late-flowering spring and winter barley (Comadran et al. 2012).

Among the four domesticated barleys harboring the haplotype H10, we found a 2-rowed, naked landrace from Turkey (FT537) that was collected by Jack Harlan in 1948. The allelic status at *HvCEN* is not known. In contrast, the haplotype combination H10–HI was found in (i) one landrace from Nepal (6-rowed, hulled); (ii) one cultivar from India (6-rowed, hulled); and (iii) one 2-rowed, naked cultivar from Japan (Fig. S7). We therefore speculate that wild barleys from Iran contributed to the genetic makeup of domesticated Asian barleys.

### Selection dynamics

Crop domestication is typically associated with a reduction of diversity in respect to the ancestral wild species, mainly due to subsampling of populations and selection of variants beneficial for cultivation. While barley is a relatively diverse crop, only a fraction of the wild diversity has passed through the domestication bottleneck on the genome-wide scale (Civáň et al. 2024). Nonetheless, the *PPD-H1* locus appears relatively diverse in cultivated genotypes. We observed 14 haplotypes at *PPD-H1* (1376 bp fragment) within the diverse GWAS panel of world-wide origin (Table S7). Further, in the Diversity panel, we found 90 haplotypes within an 898 bp fragment (Table S8). In contrast, Jones et al. (2008) observed 121 haplotypes in 266 accessions that included only 72 wild barleys. Such a high number of haplotypes in a relatively small sample set is likely due to the size of the fragment (3508 bp) resequenced by Jones et al (2008). To directly compare our findings with Jones et al. (2008), the 898 bp fragments of the 266 accessions studied in their work were aligned to the sequences of the 2057 lines of the Diversity panel. Within this aligned block, we detected 65 haplotypes in the 266 accessions, slightly less than the number of haplotypes in our Diversity panel.

Comparing nucleotide diversity between wild (*n* = 942) and domesticated barley (*n* = 1110), we observed only a 9% loss of diversity at *PPD-H1*. This is also contrary to the findings of Jones et al. (2008) that reported 22.5% loss of diversity within their 72 wild and 194 domesticated barleys. With Jones et al. (2008), we have 61 wild barley ‘accessions’ in common, representing 84% of their wild barley (Table S3; Table S4). The reduction in diversity observed in Fig. 4 is consistent with barley diversity patterns and does not seem extraordinary. While Jones et al. (2008) considered admixed accessions as wild, this likely did not contribute to the stronger bottleneck they detected. Our analysis, which included resequencing and phenotypic data, identified 20 admixed accessions previously classified as wild barley in their study. Therefore, we are confident in the robustness of our result with 90 haplotypes. The higher haplotype numbers in their study may stem from issues in SNP calling and haplotype assignment, though this requires strong evidence. Differences in sample sets and fragment sizes between our study and theirs likely account for the varying results, but this does not invalidate our findings.

### Diversity patterns

Nevertheless, recent studies (Cuesta-Marcos et al. 2010; Russell et al. 2016) reported lower haplotype numbers at *PPD-H1* even from the longer-sequences (covering full gene) and diverse samples including wild barleys than Jones et al. (2008). In our study, we used comparable numbers of wild and domesticated barleys and a smaller loss in genetic diversity was observed compared to Jones et al. (2008). One reason could be that several major *Ppd-H1* haplotypes are shared among wild and domesticated genotypes and that probably led to the observation of low nucleotide diversity change. Another reason could be that the domesticated group is a mix of (i) spring, facultative and winter types, (ii) different row-types, and (iii) caryopsis types, and was collected from a wide range of environments. Thus, diversity (= expected heterozygosity on random mating) will be pushed higher in the domesticated group as a result of the winter—spring, 2-rowed—6-rowed and other polymorphisms. There may be a general point that selection that maintains polymorphism within the domesticated group, as here, will also maintain diversity or at least reduce the loss. So, we get a reverse of the usual *pre—post-domestication* pattern displaying less diversity loss than expected. Nevertheless, overall, we observed fewer haplotypes (*N* = 27) in domesticated barley (landraces *N* = 23; cultivars: *N* = 17) compared to truly wild barley (*N* = 71).

Interestingly, recent investigation in sorghum using sequencing of the archaeological samples of wild and domesticated sorghum of different historical periods revealed that the surge in diversity occurred over time and the formation of a domestication bottleneck is probably a myth (Smith et al. 2019; Brown 2019). We observed within wild and domesticated barleys segregating sites exclusive to either group. Segregating sites exclusive to the wild barley indicates that there are many mutations in wild barleys that were possibly not selected in domesticates. However, mutations that occur exclusively in the domesticated barleys indicate that these mutations probably occurred after the initial domestication and/or outside the natural distribution range. Since wild and domesticated barleys can coexist together in the farmer’s fields, natural gene flow between both types may alter the values of genetic diversity. Such cases are reported to be relatively rare and are unlikely to be important in nature (Abdel-Ghani et al. 2004; Russell et al. 2011; Hübner et al. 2013). However, we believe that the rate of re-introgression of wild barley alleles probably occurred more frequently than previously thought.

To broaden the genetic basis for barley improvement at *PPD-H1*, haplotype information from this study could be considered. Potentially beneficial haplotypes could be introgressed from wild barley into the elite background (Dempewolf et al. 2017; Kilian et al. 2021; Parrado et al. 2024). Gene editing will provides another opportunity.

### The late-flowering allele most likely originated ‘*post*’ domestication and in the Fertile Crescent

Barley domestication is complex, with debates on whether it resulted from single or multiple events (Badr et al. 2000; Kilian et al. 2006; Morrell and Clegg 2007; Dai et al. 2012; Poets et al. 2015; Pourkheirandish et al. 2015; Pankin et al. 2018; Zeng et al. 2018; Civáň et al. 2021). A key adaptation for barley's expansion was the evolution of late-flowering ecotypes (*ppd-H1*). Two main hypotheses exist: (I) late-flowering arose *post*-domestication outside the Fertile Crescent (Turner et al. 2005; Cockram et al. 2007), or (II) it originated in wild barley from Iran before domestication (Jones et al. 2008).

Our study shows all late-flowering haplotypes derive from the wild-type H10 haplotype, and the causative mutation is exclusive to domesticated barley. Building on sequence data from previous studies (Comadran et al. 2012; Pourkheirandish et al. 2015), we propose that a natural mutation occurred in a domesticated two-rowed, facultative barley in the Southern Levant, supporting a *post*-domestication origin. One scenario suggests hybridization between domesticated barley with the wild-type allele (e.g., H4, like Masada barley) and wild barley carrying H10 in the Southern Levant (Fig. 5). Their offspring later acquired the mutation at SNP22, possibly under cultivation in irrigated fields. Alternatively, a domesticated barley already containing H10 directly mutated at SNP22.

The 6000-year-old Masada barley (Mascher et al. 2016) carried the *PPD-H1*–*HvCEN* haplotype combination H4–HIV and therefore, was not late-flowering. This combination was absent from the comprehensive collection of 2057 wild and domesticated barleys (Table S3). The phylogenetically closest haplotype combination (H4–HII) was found in wild barley from Turkey, landraces from Libya and Georgia, and cultivars from six countries. The progenitor haplotype of HIV at *HvCEN* is HII, found in wild barleys from Israel and a landrace from a Jerusalem market in 1964. H4 at *PPD-H1* was also found in 54 wild barleys from Israel, suggesting the Masada barley evolved locally rather than being introduced.

The Masada barley probably grew in the Ein Gedi oasis, about 17 km from Yoram Cave (David 2015; Fig. 5). This area, with evidence of ancient irrigation systems (Hadas 2012), supports the idea that the late-flowering mutation could have occurred under cultivation in irrigated fields. Alternatively, a wild H10 barley could have mutated and then hybridized with domesticated barley under mixed stand conditions.

Late-flowering haplotypes are found exclusively in domesticated barley and potentially appear relatively recent. Although Masada barley carried the wild-type allele, late-flowering may have emerged earlier or coexisted with wild-type barley in the past, as seen today (Table S3). Further research is needed to determine the precise timeline of late-flowering haplotype evolution.

### The role of *PPD-H1* in barley flowering time adaptation

The heading date (Hd) analysis in multi-location trials identified the *PPD-H1* gene as a key factor in flowering time regulation, with the gene promoting early flowering under extended day lengths. GWAS results highlighted *PPD-H1* along with other genes like *HvCEN* and *Vrn-H2*, revealing genotype-environment interactions that influence flowering time across different latitudes (Bustos-Korts et al. 2019). The detection of *PPD-H1*'s role in flowering time and latitude dependent influence is consistent with multiple studies (Alqudah et al. 2014; Muñoz-Amatriaín et al. 2014; Weigmann et al. 2019).

## Conclusion

We showed that the mutated late-flowering *PPD-H1* allele that has facilitated the cultivation of spring barley in Northern environments originated from Desert-type wild barley in the Southern Levant *post*-domestication and involved the selection of one de novo mutation (SNP22). Late-flowering haplotypes H1 and H2 increased in frequency during the spread of civilization out of the Fertile Crescent toward Northern Europe under selection pressure. Haplotype H2 probably originated also de novo (synonymous substitution) during this range extension. Further studies are needed to investigate the nature of substitutions and their potential roles in the expression and function of *PPD-H1*. This could be performed using representative samples from each haplotype found in the study.

## Supplementary Information

Below is the link to the electronic supplementary material.

Supplementary file1 (DOCX 48491 KB)

Supplementary file2 (XLSX 24 KB)

Supplementary file3 (XLSX 17 KB)

Supplementary file4 (XLSX 608 KB)

Supplementary file5 (XLSX 45 KB)

Supplementary file6 (XLSX 16 KB)

Supplementary file7 (XLSX 179 KB)

Supplementary file8 (XLSX 19 KB)

Supplementary file9 (XLSX 25 KB)

Supplementary file10 (XLSX 12 KB)

Supplementary file11 (XLSX 770 KB)

Supplementary file12 (XLSX 177 KB)

Supplementary file13 (XLSX 173 KB)

Supplementary file14 (XLSX 17 KB)

Supplementary file15 (XLSX 18 KB)

## Data Availability

New sequence data from this article are deposited in GenBank Data library under accession numbers provided in Tables S14 and Table S15: KF309068-309171.
