# Supplementary material for: On the origin of the late-flowering ppd-H1 allele in barley
Source: Theor Appl Genet. 2025 Sep 10;138(10):246. doi: 10.1007/s00122-025-04981-1 (PMC12423146; doi:10.1007/s00122-025-04981-1)
Supplement: Supplementary file 1 — Supplementary file1 (DOCX 48491 KB) [file 122_2025_4981_MOESM1_ESM.docx]

**Supplementary File 1**

# **On the origin of the late-flowering *ppd-H1* allele in barley**

Rajiv Sharma^1,2*^, Salar Shaaf^1,3^, Kerstin Neumann^1^, Petar Civan, Yu Guo^1^, Martin Mascher^1,4^, Michal David^5^, Adnan Al-Yassin^6,7^, Hakan Özkan^8^, Tom Blake^9^, Sariel Hübner^10^, Nora P. Castañeda-Álvarez^11^, Stefania Grando^6,12^, Salvatore Ceccarelli^6,12^, Michael Baum^6^, Andreas Graner^1^, George Coupland^13^, Klaus Pillen^14^, Ehud Weiss^5^, Ian J Mackay^2^, Wayne Powell^2^, Benjamin Kilian^1,11*^

^1^Leibniz Institute of Plant Genetics and Crop Research (IPK), 06466 Gatersleben, Germany

^2^Scotland’s Rural College (SRUC), Edinburgh, EH93JG, UK

^3^University of Milan, DiSAA, Via Celoria 2, 20133 Milan, Italy

^4^UCA-INRAE UMR 1095, GDEC, Clermont Ferrand, France

^5^German Centre for Integrative Biodiversity Research (iDiv) Halle-Jena-Leipzig, Leipzig, Germany

^6^The Martin (Szusz) Department of Land of Israel Studies and Archaeology, Bar Ilan University, Ramat-Gan, 5290002, Israel

^7^The International Center for Agricultural Research in the Dry Areas - ICARDA, Jordan

^8^National Agricultural Research Center (NARC), Amman, Jordan

^9^Department of Field Crops, Faculty of Agriculture, University of Çukurova, 01330, Adana, Turkey

^10^Department of Plant Sciences & Plant Pathology, Montana State University, Montana, USA

^11^Galilee Research Institute (MIGAL), Tel-Hai College, 12210 Upper Galilee, Israel

^12^Global Crop Diversity Trust, 53113 Bonn, Germany

^13^Freelance consultant, Italy

^14^Department of Plant Developmental Biology, Max Planck Institute for Plant Breeding Research, 50829 Cologne, Germany

^15^Institute of Agricultural and Nutritional Sciences, Martin-Luther-University Halle-Wittenberg, 06120 Halle/Saale, Germany

***Correspondence**:

Rajiv Sharma: [Rajiv.Sharma@sruc.ac.uk](mailto:Rajiv.Sharma@sruc.ac.uk)

Benjamin Kilian: [benjamin.kilian@croptrust.org](mailto:benjamin.kilian@croptrust.org)

**Table of Content**

**Supplementary Materials and Methods 3-6
Supplementary Results 7-12
Supplementary Figures 13-31
Supplementary References** **32**

**Supplementary Materials and Methods**

## **Multi-location field trials (GWAS panel)**

All field trial Hd data were manually checked and outliers were removed based on Z-test scores >3.5, accounting for two to five observations per trial. Restricted Maximum Likelihood (REML) was used to obtain Best Linear Unbiased Estimates (BLUEs) of mean from each location as implemented in Genstat 18 (Payne 2009). Summary statistics of Hd and heritability were calculated using Genstat. One hundred and seventy-seven accessions with complete data sets across all locations were used for GWAS (Table S1).

## **Markers for GWAS analysis**

Genome wide association studies were performed in TASSEL version 5.2.54 using MLM model (Zhang et al. 2010). A–log_10_ (P-value) = 4 was used as a significance threshold to control false positives across the GWAS. Allelic effects, which in TASSEL are calculated based on alphabetical order, were converted relative to the genotype BCC1391 due to no missing genotypic data. A linear regression model was fitted in R (www.R-project.org/) to estimate Site x SNP interaction, without population structure control from significant SNP markers.

## **Geo-referenced Diversity panel for allele mining and phylogenetic analysis**

Wild barleys from the following countries were considered, from west to east: Libya (N=2), Greece (N=4), Cyprus (N=4), Israel (N=363), Jordan (N=18), Lebanon (N=8), Turkey (N=362), Syria (N=37), Iraq (N=29), Iran (N=91), Afghanistan (N=7), Turkmenistan (N=8), Uzbekistan (N=4), Tajikistan (N=4) and ‘Former Soviet Union’ (N=1).

Based on morphological and taxonomical characterization under field conditions at IPK in Germany, 138 samples were not considered for allele mining (Table S4). Among them were 128 samples formerly assigned as *Hordeum spontaneum* (based on genebank passport data), but all these samples showed non-wild characteristics - based on morphology (non-brittle/ non-shattering types, no awns, naked caryopsis), or gene sequence data at *Btr1* and *Btr2* loci (Pourkheirandish et al. 2015) and at seven other nuclear loci (Jakob et al. 2014) (Table S4).

## **DNA Amplification and re-sequencing at *PPD-H1***

The Primer3 online software (http://bioinfo.ut.ee/primer3-0.4.0/; Untergasser et al. 2012) was used to design two primer pairs for PCR amplification and re-sequencing. Both primer pairs amplified in total 1428 bp of *PPD-H1* (positions 12960 – 14388 of cultivar Morex, AY943294).

The first primer pair (P5F-P5R, Jones et al. 2008) covered SNP48 located in exon 6 and amplified 619 bp of cultivar Morex (positions 12960 to 13579): P5F (forward) 5’- GATGGATTCAAAGGCAAGGA-3’ (12960 to 12976 of AY943294) and P5R (reverse) 5’- CGTTAGAGCCCTGCTTCATC -3’ (13560 to 13579 of AY943294). The second primer pair (PP05-PP04, Jakob et al. 2014) covered the CCT domain encompassing non-synonymous SNP22 of Turner et al. (2005). PP05 (forward) 5’-GTGCAAAGCATAATATCAGTGTCC-3’ (13376 to 13399 of AY943294) and PP04 (reverse) 5’-GGCCAAAGACACAAGAATCAG (14368 to 14388 of AY943294) amplified 1012 bp (positions 13376 to 14388; Turner et al. 2005).

The GWAS panel was re-sequenced using both primer combinations. A 1367 bp fragment was considered for analysis. The Diversity panel was re-sequenced using the second primer combination only (PP05-PP04). After trimming, a fragment of 898 bp was considered for multiple sequence alignments.

## **Geographical distribution maps**

Maps were generated from the geographical coordinates of the original accessions. All coordinates were manually checked and standardized in decimal format. A country mismatch test was performed. This test consisted in mapping the coordinates and comparing whether the records were mapped robustly in the countries of origin reported in the passport data. Records where the country origin did not match with the country where the coordinate was mapped, were discarded. Maps are projected using the World Robinson Projection, a projection that minimizes all types of distortion over the sections of a map. Land cover data with shaded relief and country administrative boundaries maps were made with Natural Earth (naturalearthdata.com). We extracted elevation data for each geo-referenced record using CIAT’s SRTM (<http://srtm.csi.cgiar.org/srtmdata/>) resampled raster data (1 sq. m at the equator).

**Experiments to phenotypically characterize wild-type and late-flowering genotypes**

### *Hd of the GWAS panel under controlled long and short-day greenhouse conditions at IPK, Germany*

Two seeds of each genotype were sown per turf tray on August 30^th^ of 2017 and thinned out to one plant per pot after seedling emergence. To control the effect of vernalization on flowering, trays were subjected to vernalization at 4°C with an 8h light period twelve days after sowing for a period of 46 days. Afterwards, plants were transplanted to bigger pots. Three plants of each genotype were grown under long day conditions in a greenhouse with 16h light duration and 20°C/16°C day/night. In parallel, three plants of each genotype were grown under short day conditions in a climate chamber (8h light, 20°C/16°C). Heading date (BBCH55) was recorded on the main tiller of each plant. Within each treatment, plants were grown in fully randomized design. Phenotypic data was manually curated and outlier test was performed. ANOVA, heritability and Best linear unbiased estimates (BLUEs) were calculated. Genotypes and their haplotypes considered for this study are indicated in Table S3.

### *Heading date under vernalized and non-vernalized long-day field conditions at IPK*

Seeds were sown in turf trays at day 56 (vernalized treatment) and at day 99 (non-vernalized treatment) of the Julian calendar in 2010. Plants of the vernalized treatment received 43 days of vernalization in a climate chamber with 8h light and 4°C constant temperature. The experiment was conducted with two treatments (vernalized and non-vernalized) each with 2 replications. In each replication, 2 plants from each genotype were considered (in total 4 plants per genotype per treatment). Plants of both treatments (similar developmental stage) were transplanted to the field at day 119 of the Julian calendar (30^th^ of April) to avoid an effect of potential natural vernalization under field conditions in Germany. In the vernalized treatment, the following traits were scored: date of physiological maturity, growth habit (prostrate, intermedium and erect-type), plant height, flag leaf width, ear length and ear width. Outliers, repeatability and BLUEs were calculated as reported earlier.

## **Analysis of Environmental Data**

We used Principal Component Analysis (PCA) to rank the contribution of each bioclimatic variable. PCA was conducted using the R package FactoMiner (Le et al. 2008).

To infer environmental clusters of 1375 collection sites, Discriminant Analysis of Principal Components (DAPC) using the R package Adegent was employed (Jombart et al. 2010). The prior number of clusters were set to 2 to 20. Intra-correlated variables with high correlations exceeding r=0.9 were removed to allow spatial resolution. A total of 15 different indexes were used to test the possible number of clusters.

## **Supplementary Results**

***Adaptation of barley depends upon the environment***

The region near the *Vrn-H3*/ *HvFT1* gene showed strong peaks at three sites (Germany, Turkey, USA). This is interesting as it was reported that *HvFT1* is the central regulator where signals are perceived that promote flowering (Nitcher et al. 2013). It is not known why no association was detected in this genomic region in Syria. Either it suggests that different loci in high linkage disequilibrium (LD) but in dispersion contribute to the effects observed in our study or that contrasting alleles within the gene cause these effects which could be supported by observing late heading effects in Syria. Further study is needed to ascertain the effects from the *Vrn-H3*/ *HvFT1* locus by re-sequencing the region. It has been shown recently that at least four identical copies of the *Vrn-H3*/ *HvFT1* gene are present in barley varieties that have spring growth habit whereas a single copy is present in most other barley varieties. Therefore, copy number variation causes huge effects on the expression of *Vrn-H3*/*HvFT1* (Nitcher et al. 2013) and may explain the variation seen in this region. Re-sequencing could shed more light on the observed differences. As shown by Casas et al. (2011), four HvFT1 haplotypes contributed to differences in flowering time but at the SNP level we could not ascertain the differences precisely.

Our findings confirm Maurer et al. (2015) and Herzig et al. (2018) that under European long-day conditions (Scotland, Germany) lines of the Nested Association Mapping (NAM) population HEB-25 carrying wild-type alleles flowered substantially earlier than barley carrying the late-flowering allele.

In a later study with selected HEB-25 lines segregating for wild-type and late-flowering alleles, the early flowering effect of *Ppd-H1* alleles was verified in Scotland, Germany and Jordan), but disappeared under field conditions in the United Arab Emirates and Australia with Hd at day lengths below 13 hours (Wiegmann et al. 2019). The drought conditions in Al-Karak (Jordan) lead to a positive effect of the *Ppd-H1* wild-type allele on grain yield.

Further study could ascertain the environment-specific effects of *PPD-H1*. A similar effect as in Al-Karak can be expected in Israel, where the Desert type of wild barley with haplotype 10 was found. In this region, where barleys flower early (before Easter; Passover = Easter is the holyday of the barley harvest), the difference in heading date between haplotypes that respond to the photoperiod, but also between wild-type and late-flowering haplotypes could be much less pronounced than in higher latitudes.

## ***Genetic diversity at PPD-H1 within the Diversity panel***

Wild barleys from Israel possessed the highest genetic diversity (47 haplotypes), followed by Turkey (19 haplotypes). The number of haplotypes mostly decreases from Israel to the west: Israel (N=47), Cyprus (N=3), Greece (N=2) and Libya (N=1), and from Israel to the east: Israel (N=47), Jordan (N=9), Lebanon (N=4), Syria (N=8), Turkey (N=19), Iraq (N=2), Iran (N=5), Afghanistan (N=2), Turkmenistan (N=1), Uzbekistan (N=1) and Tajikistan (N=1). Several haplotypes were region-specific: for example, H34, H39 and H66 were specific to Israel.

Sixty-three haplotypes were unique to wild barley and not exploited in the domesticated barleys, which might harbor a key to local environment adaptation (Table S3). Interestingly, several of the non-exploited haplotypes clustered together, like (i) H48, H36, H38, H39, H85, H28, H5, H29, or (ii) H16, H62, H78, H34, H46 and H67 (Fig. 4). Moreover, these haplotypes were distinct and differed from the closest major haplotypes by several base pairs. For example, H48 differed from H7 by 10 SNPs or H62 by 9 SNPs from H4 (Fig. 4).

## ***Linkage disequilibrium around the PPD-H1 genomic region***

We investigated the LD blocks in neighboring areas and the *PPD-H1* region, revealing a high LD region around PpdH1 that indicates a genetic bottleneck in the domesticated gene pool (Fig. S16).

Our observation aligns with the findings of Russell et al. (2016), who reported no significant difference in haplotype diversity for *HvPPD-H1* between wild and cultivated barley in a collection of 267 wild and cultivated barley accessions.

## ***Examples about the geographic distribution of wild-type haplotypes***

**(1)** H75 -> H7 -> H8 -> H92: Haplotype H7 was found in 256 genotypes of almost equal frequency in wild (13.7%) and domesticated (11.4%) barleys. Among the wilds, haplotype H7 was found in 33.3% of wild barley from Israel and in 0.6% of the genotypes from Gaziantep/Turkey. Also, 88 landraces predominantly collected in North Africa and the Near East harbored this haplotype. Haplotype H8 was found in 0.5% of wild barley (4x ISR, 1x CYP) but in 9.2 % of domesticated barley, representing mostly landraces collected in the eastern Mediterranean. Haplotype H92 was mainly found in landraces from Algeria.

**(2)** H75 -> H13 -> H4 -> H3: Haplotype H13 was detected in 13 wild barleys and one landrace barley from Chad. Haplotype H4 represents a major haplotype and was detected in 230 genotypes (14.33% of wild and 8.55% of domesticated). Wild barleys carrying H4 were collected in Greece (N=1), Israel (N=54, including the Desert-type barley FT143), Jordan (N=6), Lebanon (N=4), Syria (N=6) and Turkey (N=64). The remaining were 39 landraces mainly from the Fertile Crescent but also from Libya (N=3), and 56 cultivars predominantly from Turkey. All wild barleys harboring haplotype H3 (N=18) were collected west of Gaziantep in Turkey.

**(3)** H6 and derived haplotypes: Haplotype H6 was the most frequent haplotype and detected in 502 genotypes: 40.44% (N=381) of wild barley, 10.72% (N=119) of domesticated barley (40 landraces, 77 cultivars) and two of H. agriocrithon. The haplotype H6 was most frequent in wild barley and showed a more eastern distribution compared to the haplotype H7. The frequency of H6 increased from west to east: Israel (2.2%) - Jordan (11.1%) - Lebanon (12.5%) - Syria (54.1%) - Turkey (60.5%) - Iraq (93.1%) - Iran (90.1%) - Afghanistan (85.7%) - Turkmenistan (100%) - Uzbekistan (100%) - Tajikistan (100%). Several population/ region-specific haplotypes (all unique to Turkey) derived from H6, for example: H41, H49, H50 and H53.

## ***Analysis of environmental data***

The collection sites of wild barley from Israel containing haplotype 10 are more similar to the Masada environment than the collection sites of haplotype 10 wild barley from Iran. DAPC analysis provided complementary results. The most important variables were Bio14, Bio15, Bio18, Bio9, Bio10, and Bio19, respectively (Fig. S13). Four environmental clusters were detected (Figs. S14, S15; Table S11a-c). Cluster 1 contained 543 wild barleys, mainly from the central and eastern part of the Fertile Crescent and 194 landraces. Among the wilds were the three wild barleys from Iran harboring H10. Cluster 4 contained 505 genotypes. A total of 397 wild barley collected mainly from the Eastern Mediterranean and the Near East were assigned to this cluster (including all 363 samples from Israel, and thus all samples containing H10 from Israel. In addition, 108 landraces, mainly from Africa, the Arabian Peninsula and the Near East, as well as the Masada barley were included in cluster 4 (Table S11). Late-flowering barley was found in all four clusters.

The environmentally third closest collection site to Masada is the collection site of B1K-05 (Israel, Neomi), where FT013, FT014, FT015 and FT016 (all Desert type) were collected. All wild barleys from this population carried H66 at *PPD-H1*. FT013, FT015 and FT016 were also re-sequenced at *Btr1* and *Btr2* (Pourkheirandish et al. (2015) and were among the closest wild barleys to *btr1* (Fig. 5; Table S3).

## ***Vernalization requirement and phenotypic performance of genotypes containing haplotype H10 under long-day conditions***

In a field trial at IPK in 2010, under vernalized and non-vernalized conditions, heading date of 843 genotypes of wild and domesticated barley was investigated to determine their vernalization requirement and to characterize key agronomic traits in the vernalized treatment. For all traits, the repeatability of data was high and ranged from 0.86 to 0.98.

In the non-vernalized treatment, 582 genotypes were heading, while the remaining 261 genotypes (30%) were not flowering and therefore considered winter types.

On average, heading was reached 15 days later under non-vernalized compared to vernalized conditions among the set of 582 genotypes. Interestingly, a bimodal distribution of the heading date differences was observed, indicating two phenotypic groups - spring and facultative growth habit (Fig. S9). In group 1, there were 232 genotypes with relatively similar heading dates in both treatments, where the range of heading date difference was -7 to +14 days (average heading date difference: +5.9 days). Group 2 consisted of 350 genotypes with a delayed heading date under non-vernalized conditions and the difference ranged from 14.2 to 35.8 days (average heading-time difference: +23.9 days). Genotypes from group 1 were classified as spring (flowering without vernalization), genotypes from group 2 as facultative types (flowering without vernalization but earlier when vernalized).

Of all 843 barley genotypes, 97 wild accessions did not exhibit completely wild characteristics and were excluded from subsequent analysis, leaving 746 accessions for comparison (470 wild and 276 domesticated). Based on our experiments that depict bimodal distribution, 204 accessions were classified as spring types (6 were wild barley from Israel) and 305 as facultative, while 237 accessions were classified as winter types. A total of 70 different haplotypes were represented within this panel. To compare the haplotypes for the phenotypic key characteristics assessed in the vernalized treatment, we excluded haplotypes present in less than five genotypes, resulting in a panel of 404 wild barley and 267 domesticated genotypes covering 17 different *PPD-H1* haplotypes.

The genotypes carrying the late-flowering haplotypes H1 and H2 at *PPD-H1* were mostly spring types. In contrast, the wild-type progenitor haplotype H10 was mainly found in facultative types (wild barley from Israel, N=9; and wild barley from Iran, N=3) but also in two spring types (wild barley FT147 from Israel, landrace FT537 from Turkey) and one winter type (wild barley FT002 from Israel) (Fig. S9b). The genotypes with haplotype H10 showed a short life cycle with the second earliest heading date (1. H66, 2. H10, 3. H26) and the earliest maturity date (1. H10, 2. H66, 3. H26) of all haplotypes under vernalized, long-day field conditions in Germany (Fig. S9c; Table S9), even if only wild barley was considered (Fig. S9d). In addition, the H10-containing genotypes were among the three genotypes with the shortest plant height, narrowest flag leaves, shortest main ear and narrowest main ear width (Table S9). From these data we conclude that plants containing haplotype 10 are well adapted to their local environmental conditions in the Southern Levant or in Khuzestan, and that they are characterized by facultative or even spring growth habit.

It should be noted that genotypes containing H10 or other haplotypes found in Desert-type wild barley (e.g. H26, H66) flowered later than haplotypes H1 and H2 under long-day and non-vernalized field conditions in Germany (Table S9). This is somewhat surprising because they flower (very) early and actually under short day conditions in their native environments in the Southern Levant but confirming their facultative growth habit. The only spring type wild barley with H10 (FT147 from Israel, Havarim stream) flowered very early in both treatments.

## **Supplementary Figures
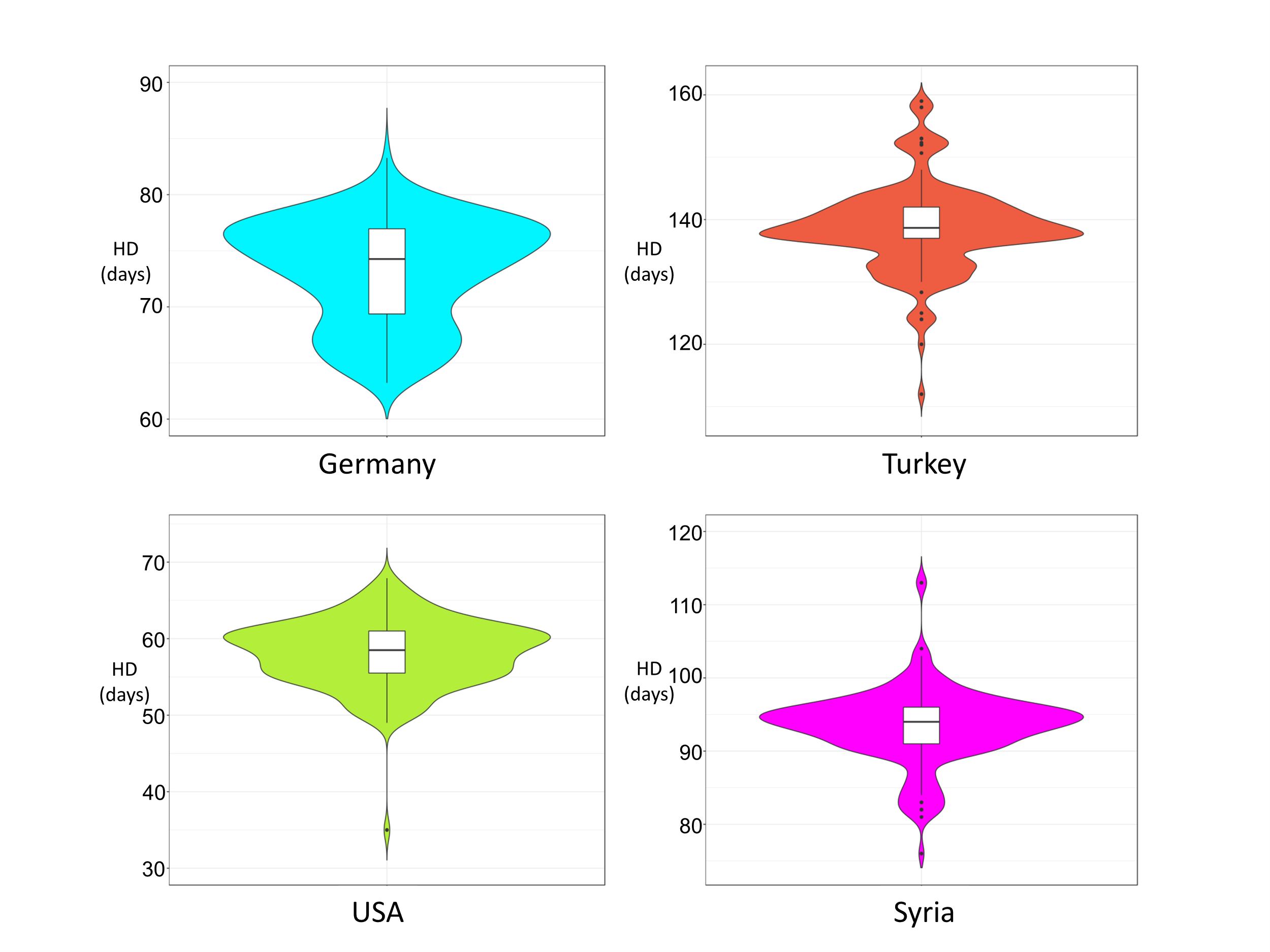
**

**Fig. S1:** Phenotypic distribution of days to heading (Hd) over four multi-location field trials. Violin plots of Hd (in days) across locations are shown. Violin plots display the distribution of heading date (Hd, in days) on the x-axis, with the names of the trial locations shown on the y-axis. Each violin represents the density and variability of Hd measurements at each location, illustrating differences in phenology across environments.


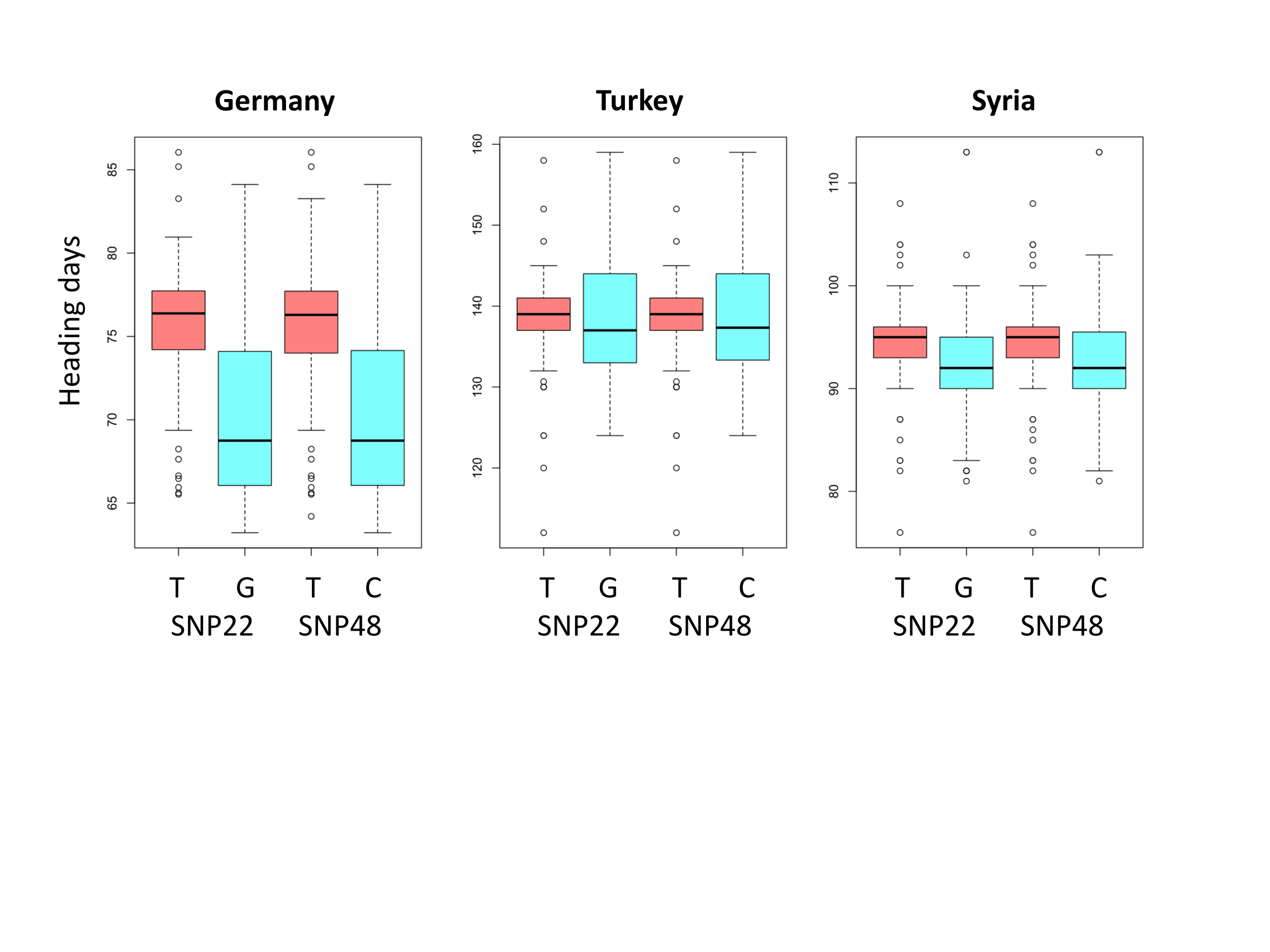


**Fig. S2:** Heading date variation across the three locations where *PPD-H1* was QTL based on the putative causal SNPs. Box plots displaying days to heading (Hd) differences in X axis considering SNP22 (T/G) and SNP48 (T/C) in Y-axis.


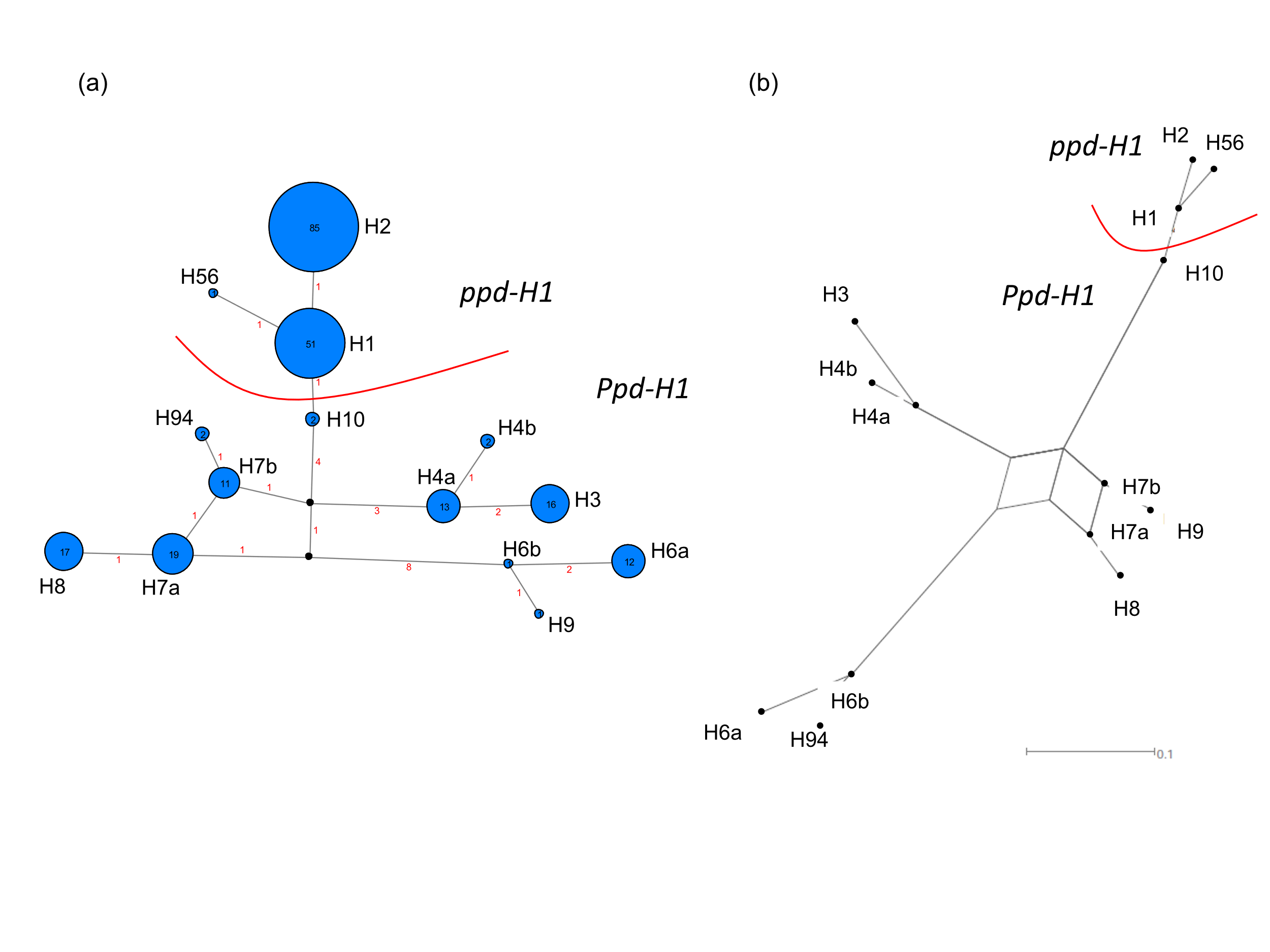


**Fig. S3:** Phylogenetic networks derived from re-sequenced *PPD-H1* fragments (1367 bp) based on the GWAS panel. (a) MJ-network (as in Fig. 4) but based on the 14 haplotypes identified in the GWAS panel (Table S3). (b) NeighborNet network computed for these 14 haplotypes.


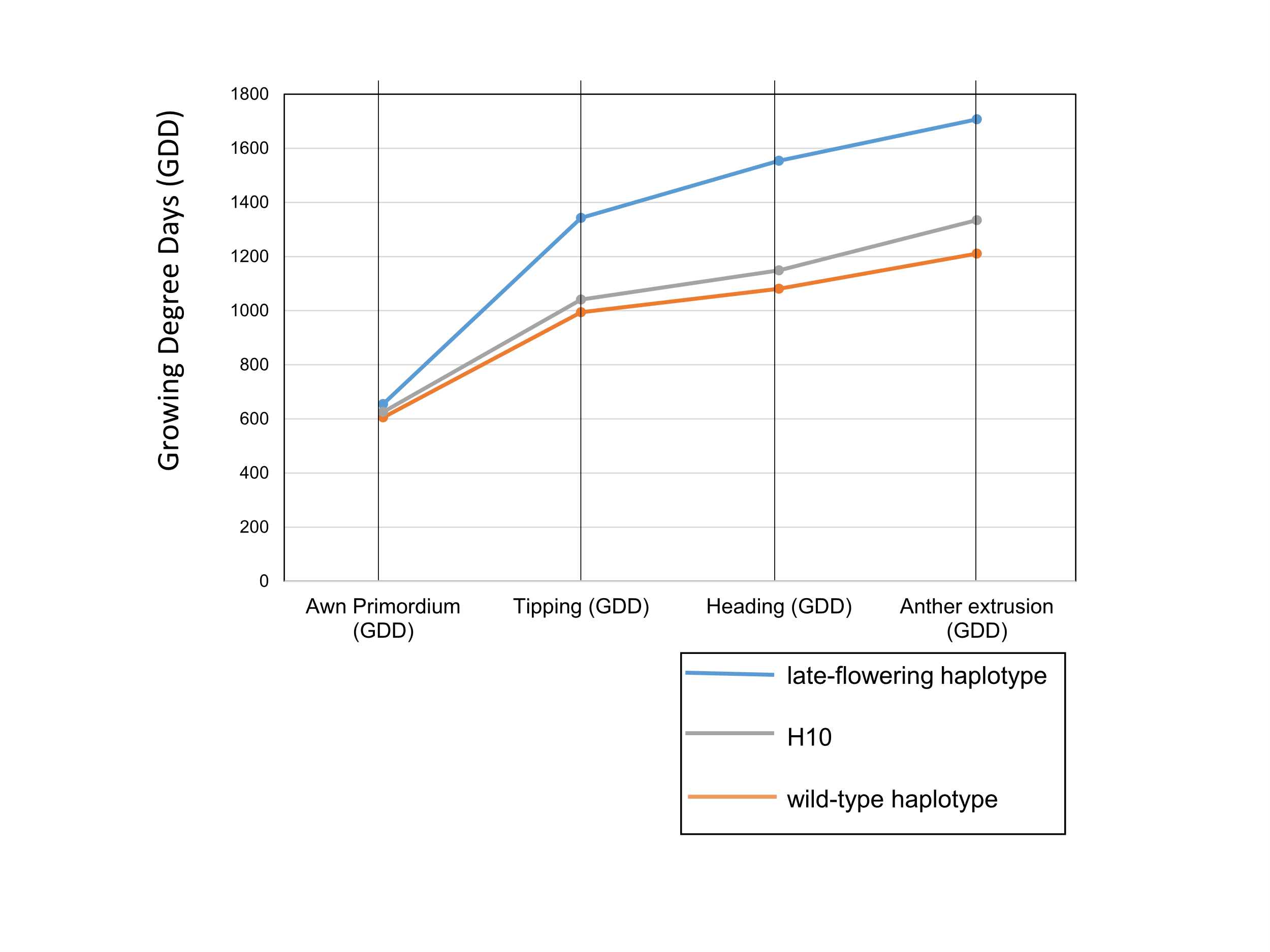


**Fig. S4:** Differential response of wild-type and late-flowering allele containing genotypes of the GWAS panel during four developmental stages under inductive long day conditions in the greenhouse at IPK. Lower Growing Degree Days (GDD) values for haplotype H10 containing genotypes (BCC533, BCC759) compared to late-flowering genotypes indicate accelerated heading dates for haplotype H10 containing genotypes (Alqudah et al. 2014). This provides further evidence that H10 containing genotypes are early flowering.

Thermal time measured as GDD from sowing to awn-primordium, tipping, heading and anther extrusion stages is provided (y-axis). Blue: late-flowering haplotype (*ppd-H1*) containing genotypes; Grey: wild-type haplotype (*Ppd-H1*) containing genotypes; Orange: H10 containing genotypes. See Figs. 2b, 4; Fig. S3; Table S3.

**
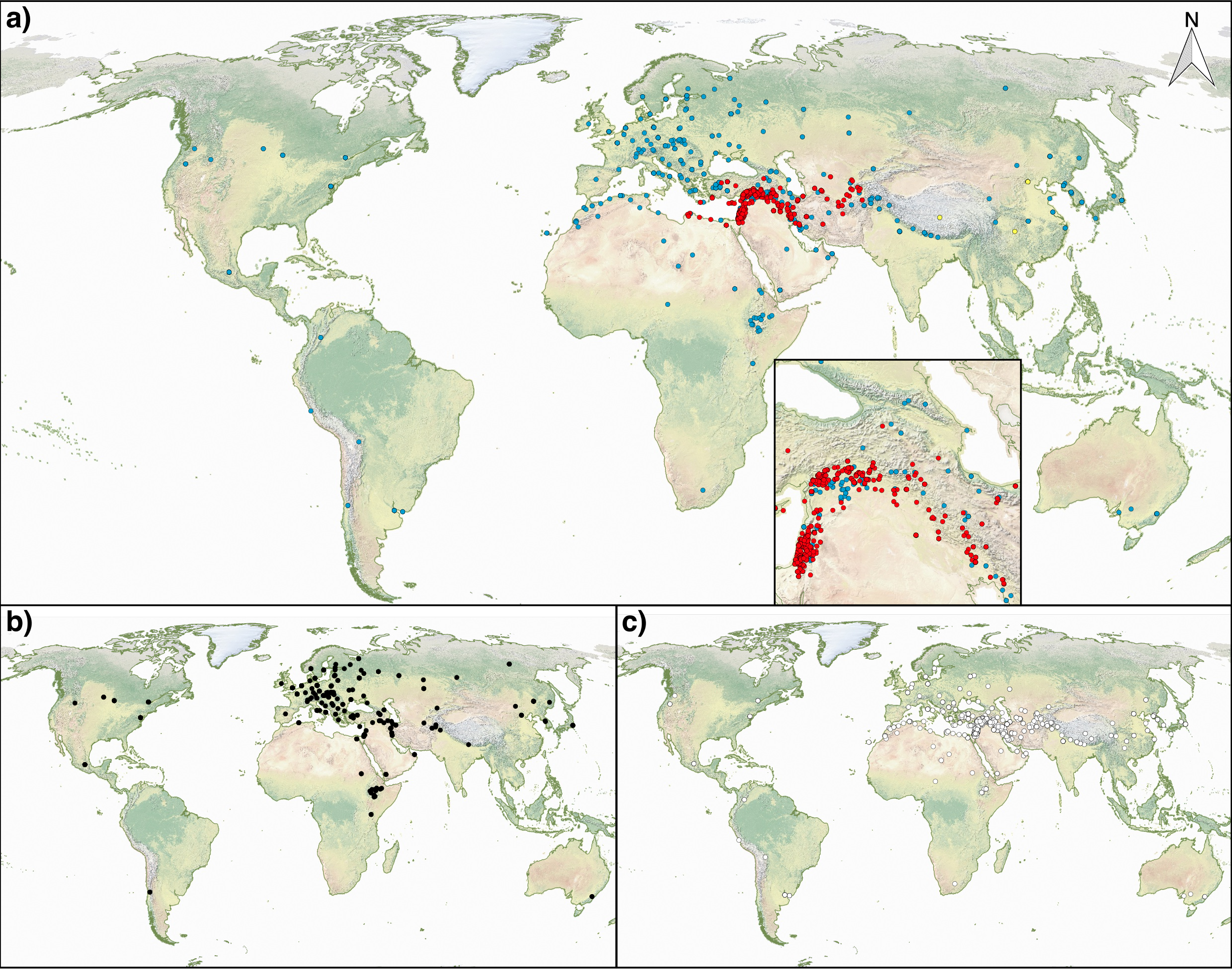
Fig. S5:** Geographical distribution of the Diversity panel. (a) Overview of the distribution of the 2057 accessions on the world-map (red = *H. spontaneum*; yellow = *H.* *agriocrithon* and blue = *H. vulgare*). The Fertile-Crescent region is enlarged; (b) geographical distribution of late-flowering, and (c) geographical distribution of wild-type haplotypes.


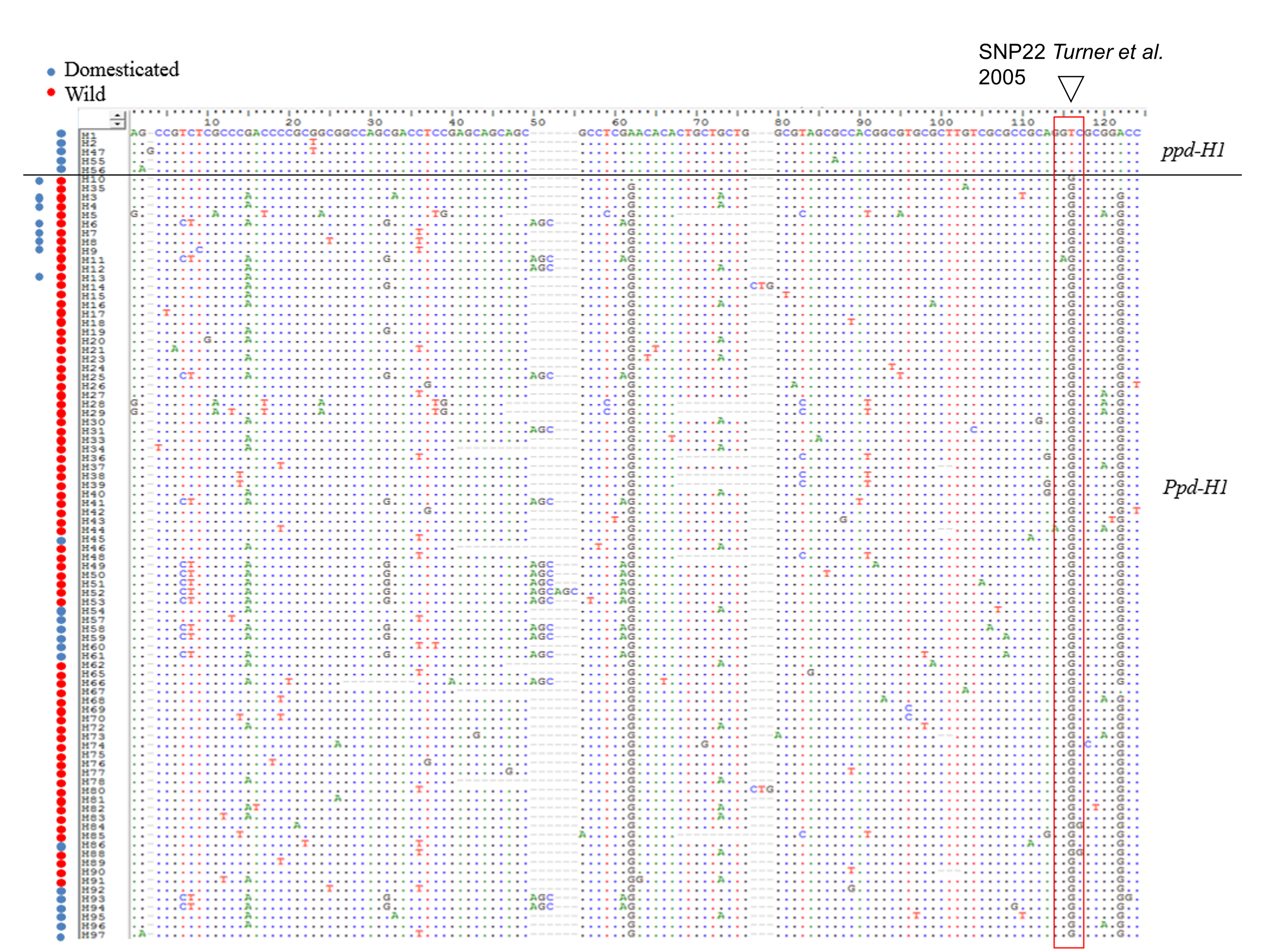


**Fig. S6:** Polymorphic sites defining the 90 haplotypes detected within 2057 re-sequenced genotypes of the Diversity panel. SNP22 is indicated by a red rectangular frame (central position, T/G SNP). Red dot on the left side, haplotype found in wild barley; dark blue dot, haplotype found in domesticated barley; dark blue and red dots, haplotype found in domesticated and wild barley.


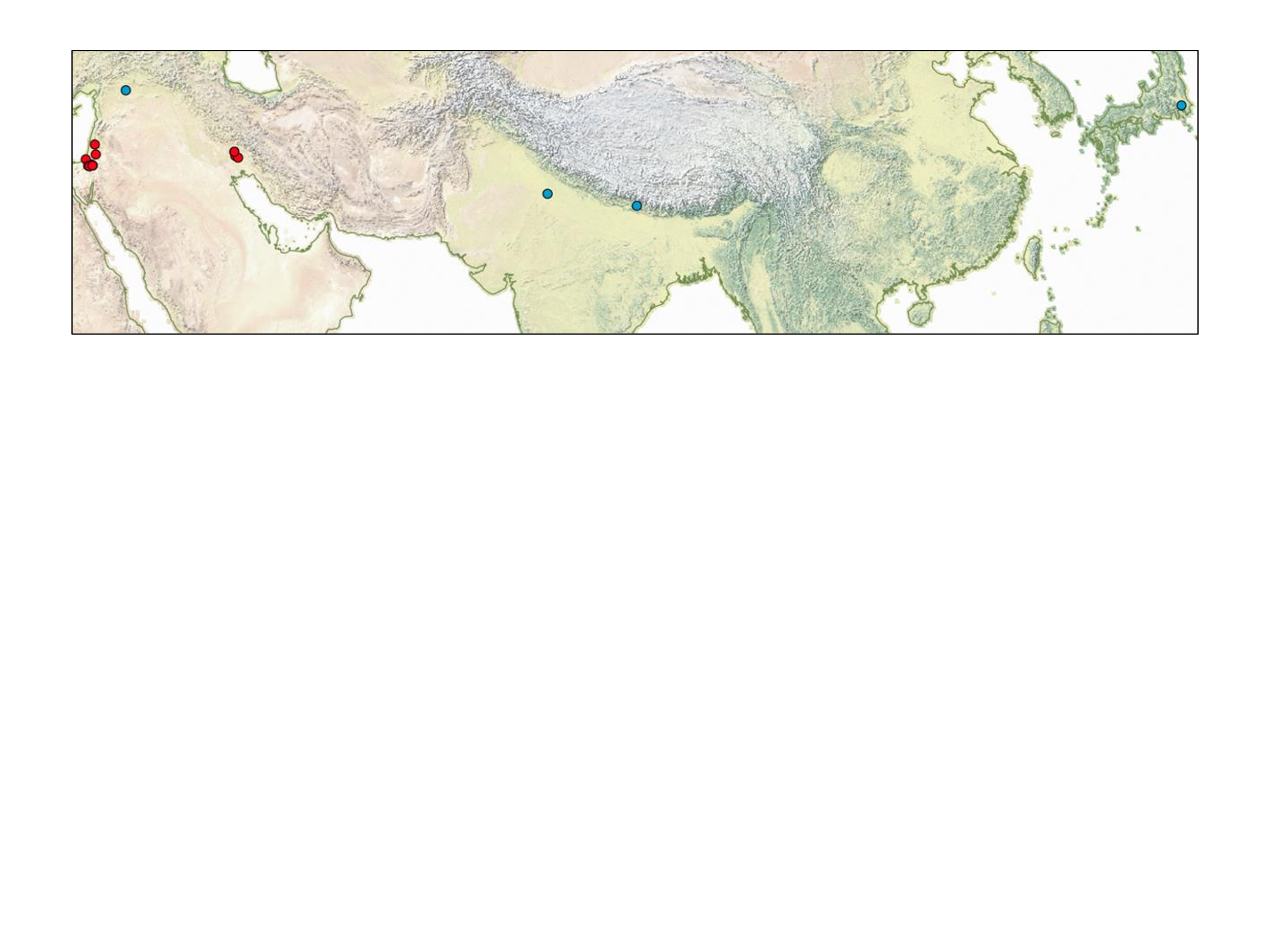


**Fig. S7:** Geographical distribution of haplotype H10 containing genotypes (red = wild barley; blue = domesticated barley). In total, haplotype H10 was found in 16 wild barleys and 4 domesticated barleys.


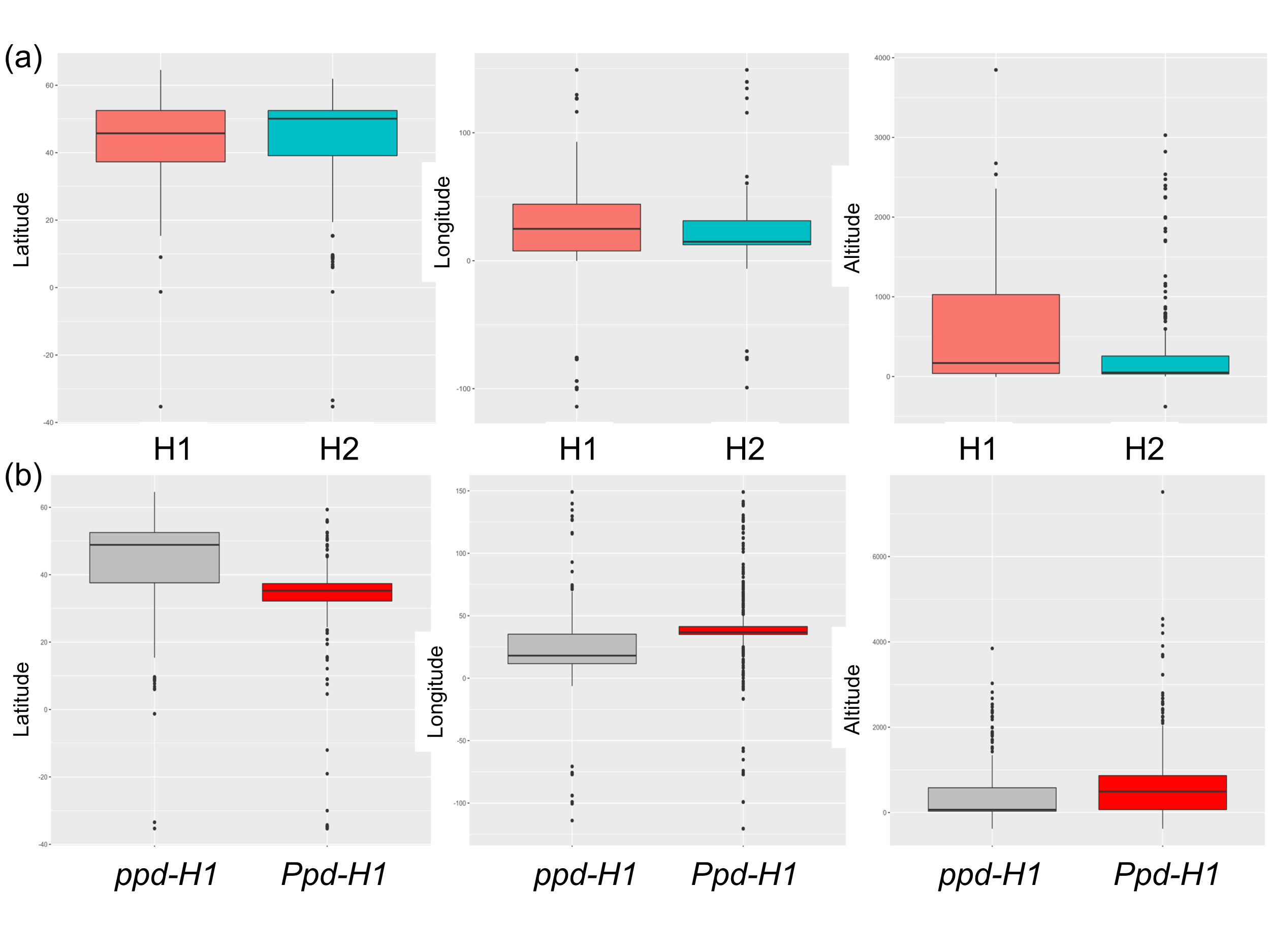


**Fig. S8:** Geographical clines of haplotypes at *PPD-H1* presented as boxplots. Comparison of latitude, longitude and altitude between (a) genotypes containing late-flowering haplotypes (H1 and H2), with the x-axis showing the late-flowering haplotypes H1 and H2, and the y-axis representing latitude, longitude, and altitude; and (b) late-flowering *vs.* wild-type genotypes , with the x-axis displaying PPD alleles and the y-axis showing Attitude, Longitude, and Latitude.


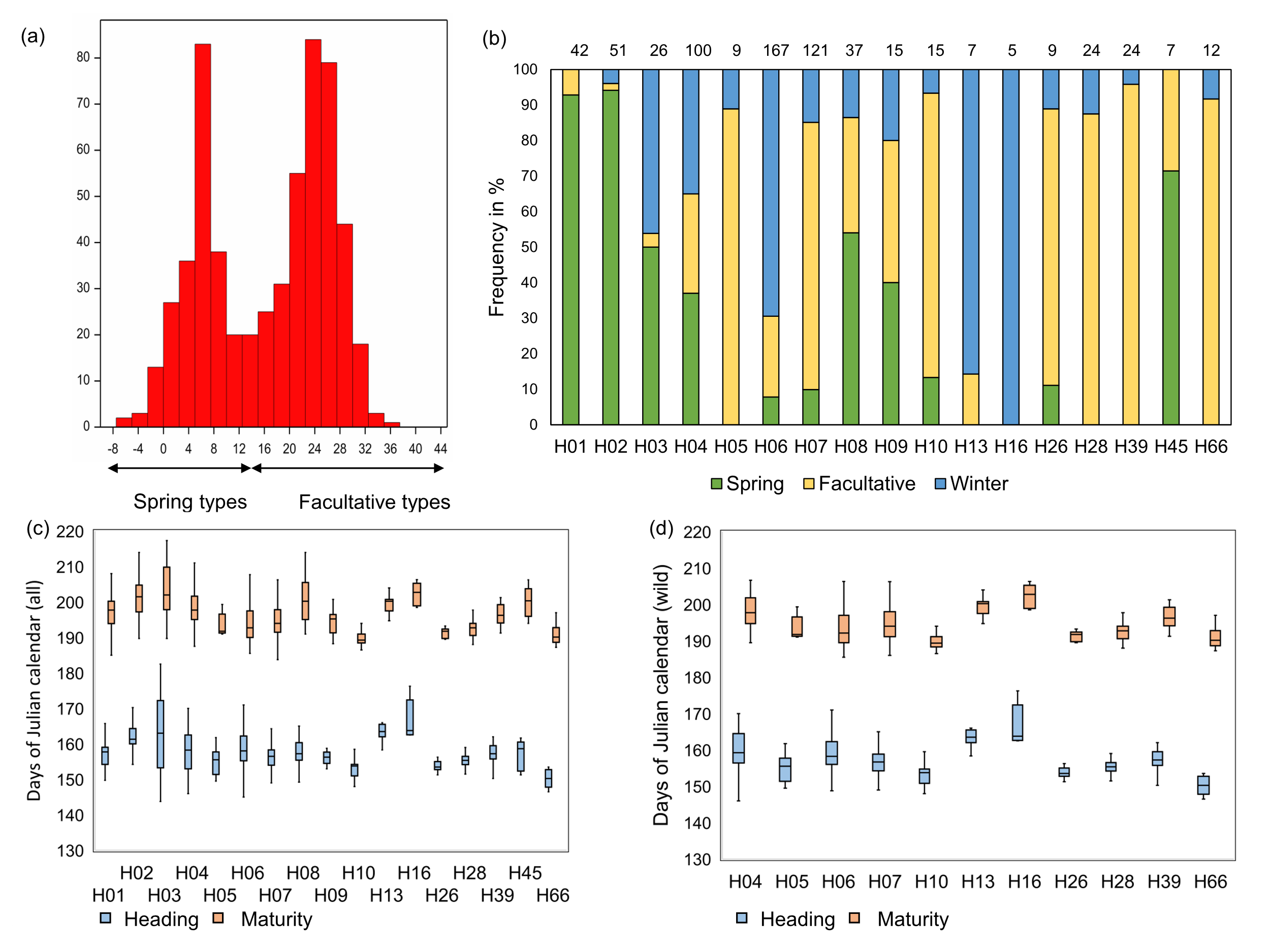


**Fig. S9:** Evaluation of vernalization requirement and phenology for 843 domesticated and wild barley genotypes of the Diversity panel grown under vernalized and non-vernalized field conditions at IPK. **(a)** Histogram of heading date differences between non-vernalized and vernalized conditions (heading date in non-vernalized minus heading date in vernalized conditions displayed in x-axis and frequency in y-axis) for 585 genotypes that were flowering in both treatments. The rest of the panel was classified as winter type (not flowered under non-vernalized conditions). **(b)** Overview of the frequency of the classified growth habit type among 17 *PPD-H1* haplotypes carried by at least 5 genotypes (in total 671 genotypes). The numbers on top refer to the number of genotypes that carry the corresponding haplotype. **(c)** Time to heading and to maturity in the vernalized treatment in wild and domesticated barley for 17 *PPD-H1* haplotypes carried by at least 5 genotypes (in total 671 genotypes. **(d)** Time to heading and to maturity in the vernalized treatment in wild barley for 11 *PPD-H1* haplotypes carried by at least 5 genotypes (in total 396 genotypes). See Table S3 and Table S4 for more information.


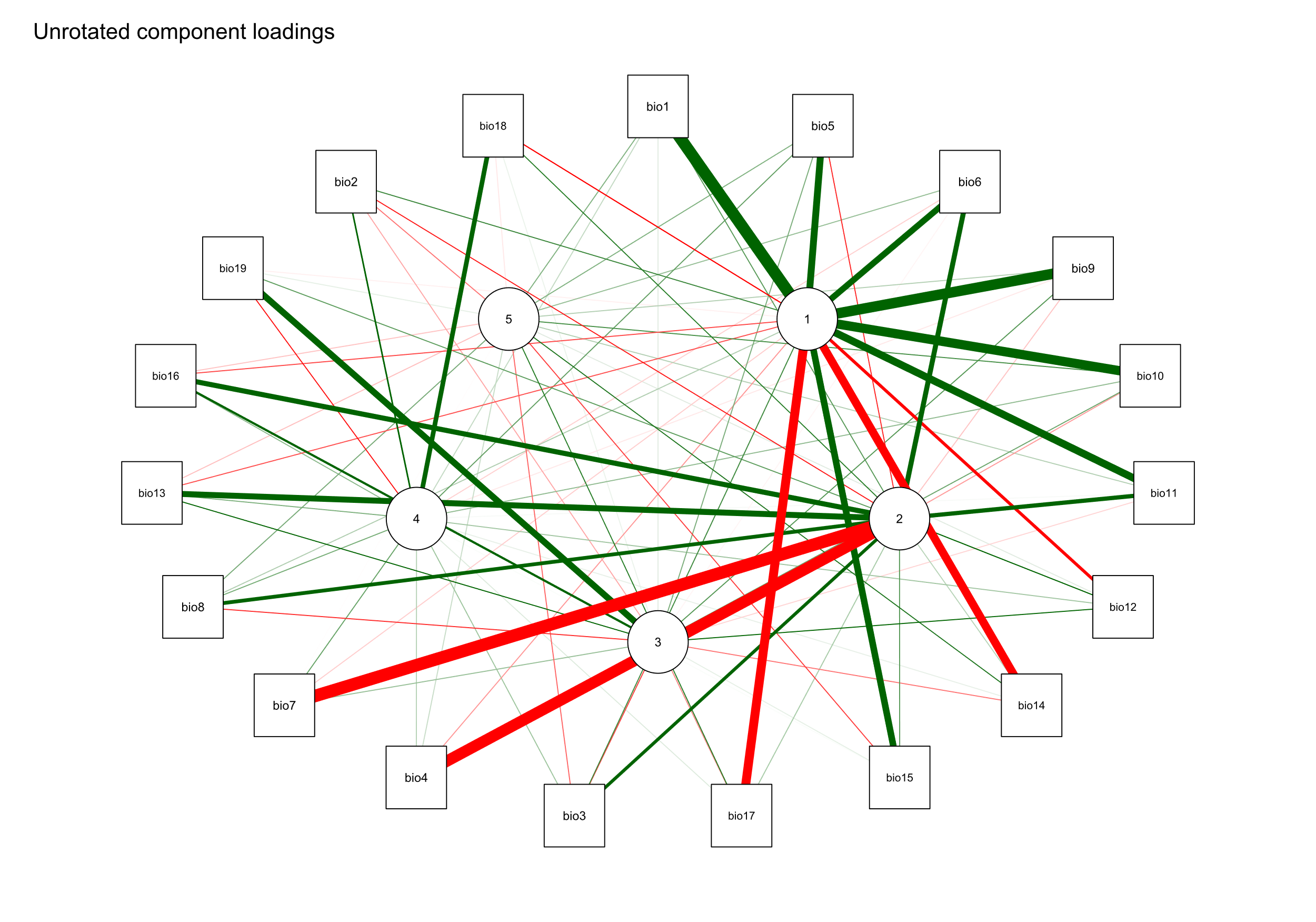


**Fig. S10:** Visualization of the first five principal component scores and their relationship with the original bioclimatic variables. A bolded vector indicates a strong positive (green) or negative (red) correlation of the original bioclimatic variable to the respective PC score.


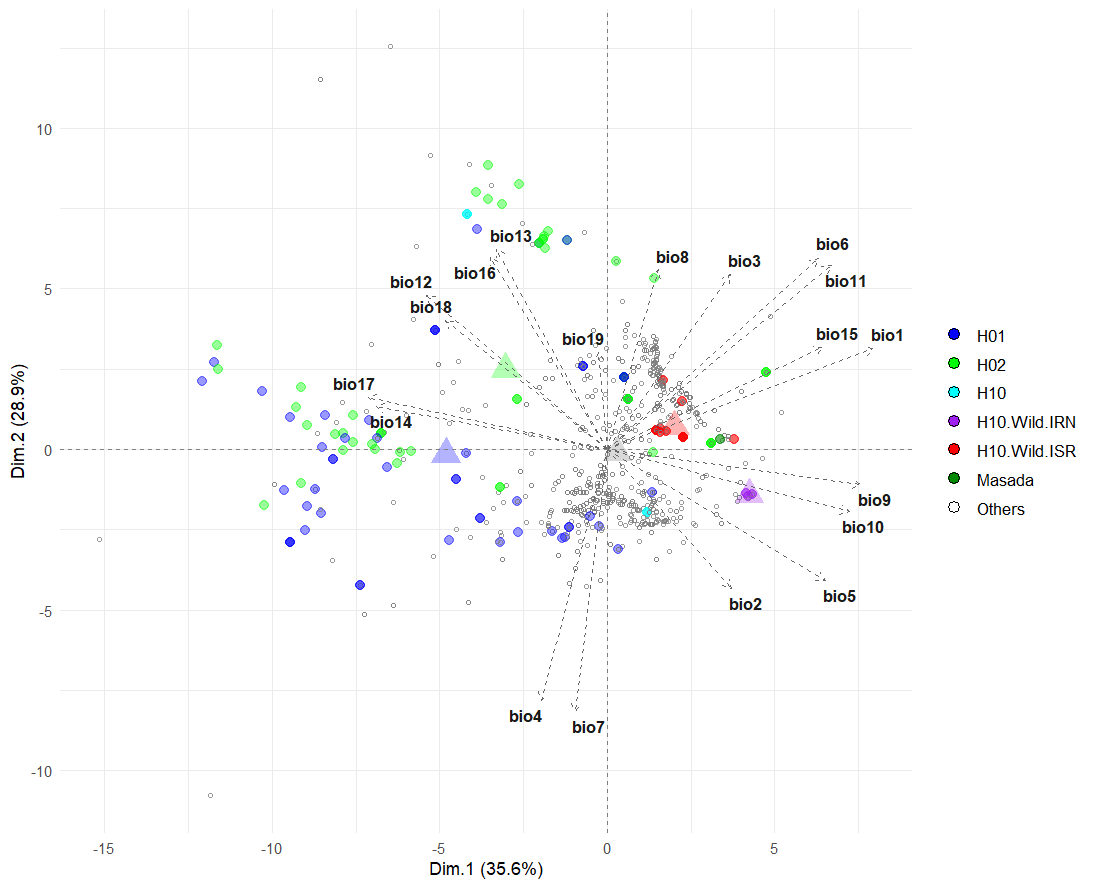
(a)


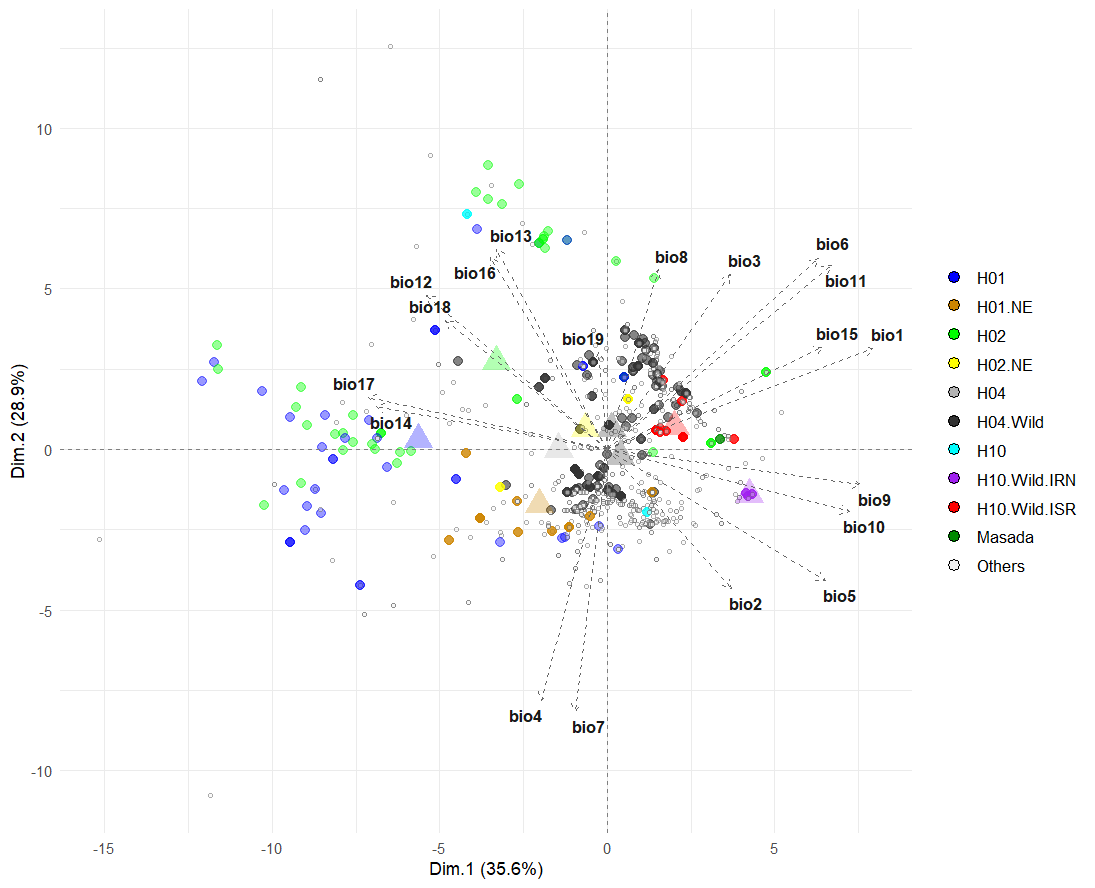


b)

c)


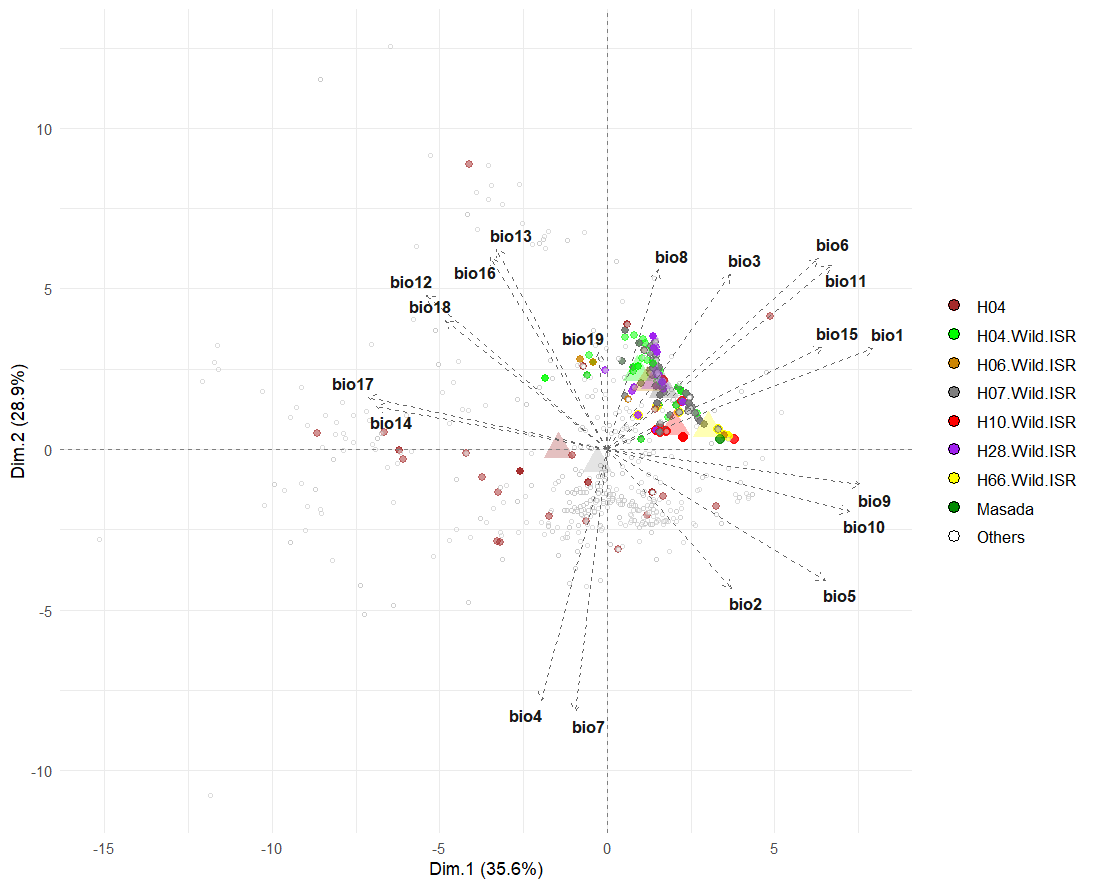


**Fig. S11:** Principal component analysis (PCA) biplot of bioclimatic variables for collection sites of 1375 genotypes (942 wild and 432 landrace barleys, and the Masada sample). **a)** late-flowering (H1, H2) and wild-type groups are separated. Two groups of H2 collection sites are visible. H10.Wild.Israel: wild barley from Israel containing haplotype; H10.Wild.Iran: wild barley from Iran containing haplotype 10. **b)** collection site characteristics of late-flowering barley (.NE – landraces from the Near East) and H4 containing landrace barley are highlighted. **c)** Zooming in on the characteristics of the collection sites of extant wild barley from Israel compared to the collection sites of the Masada sample and landraces containing H4. Wild barley containing six haplotypes are indicated. Large circles indicate the median PC projection for each haplotype. See Tables S11, S11 and Figure S10 for more information.

**
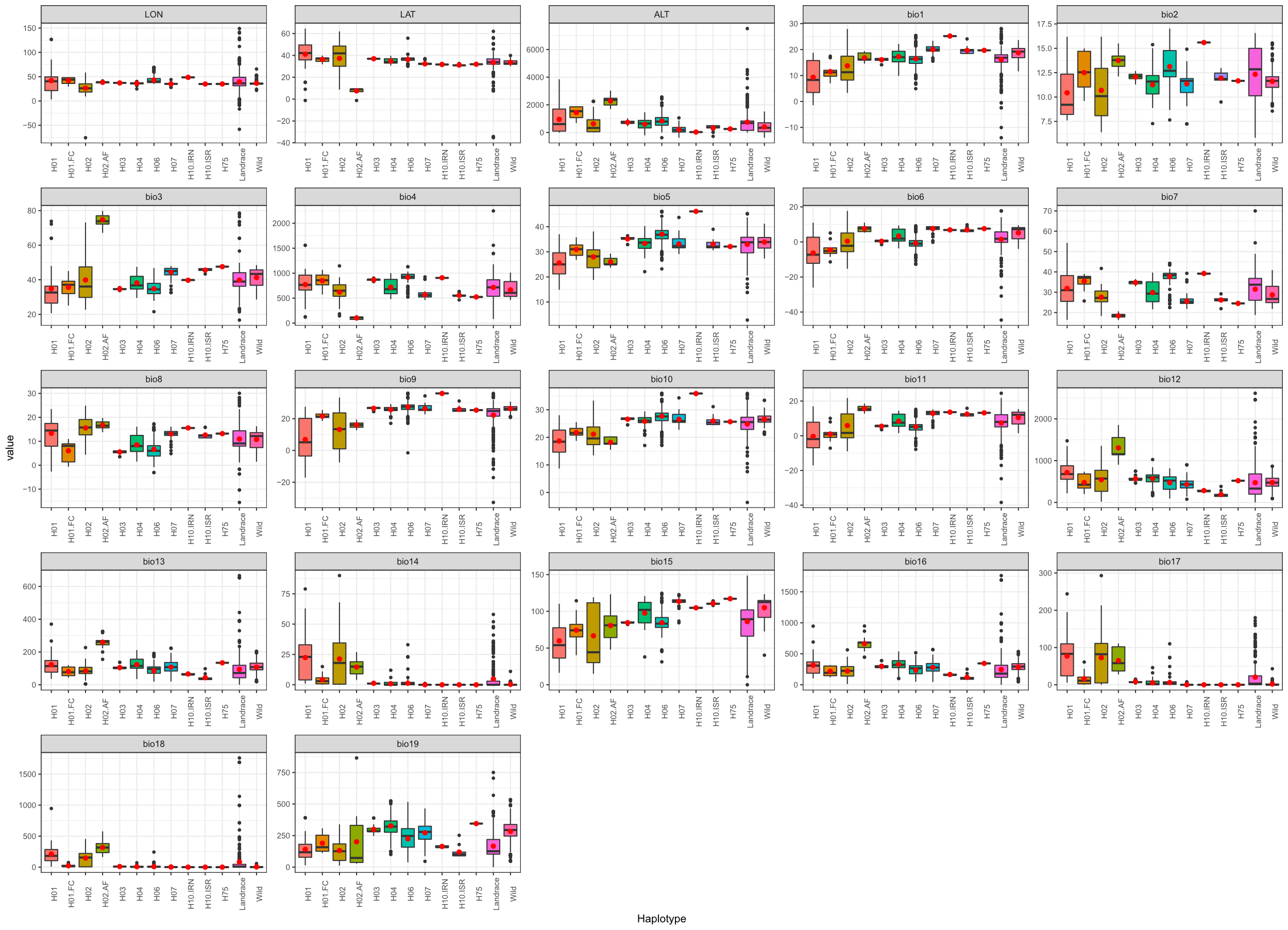
**

**Fig. S12:** Box plots of geographical and bioclimatic variables for the environmental conditions at collection sites of major *PPD-H1* haplotypes (H1, H2, H3, H4, H6, H7) and haplotypes H10 and H75, for wild and landrace barley (Lon, Lat, Alt, Bio1-Bio19). The vertical bar inside the box plot is the median value and the red dot indicates the mean value. H01.FC: H1 containing late-flowering barley from the Fertile Crescent; H02.AF: H2 containing late-flowering barley from Africa; H10.IRN: H10 containing wild barley from Iran; H10.ISR: H10 containing wild barley from Israel. Landraces: Landraces with remaining haplotypes; Wild: wild barley with remaining haplotypes.


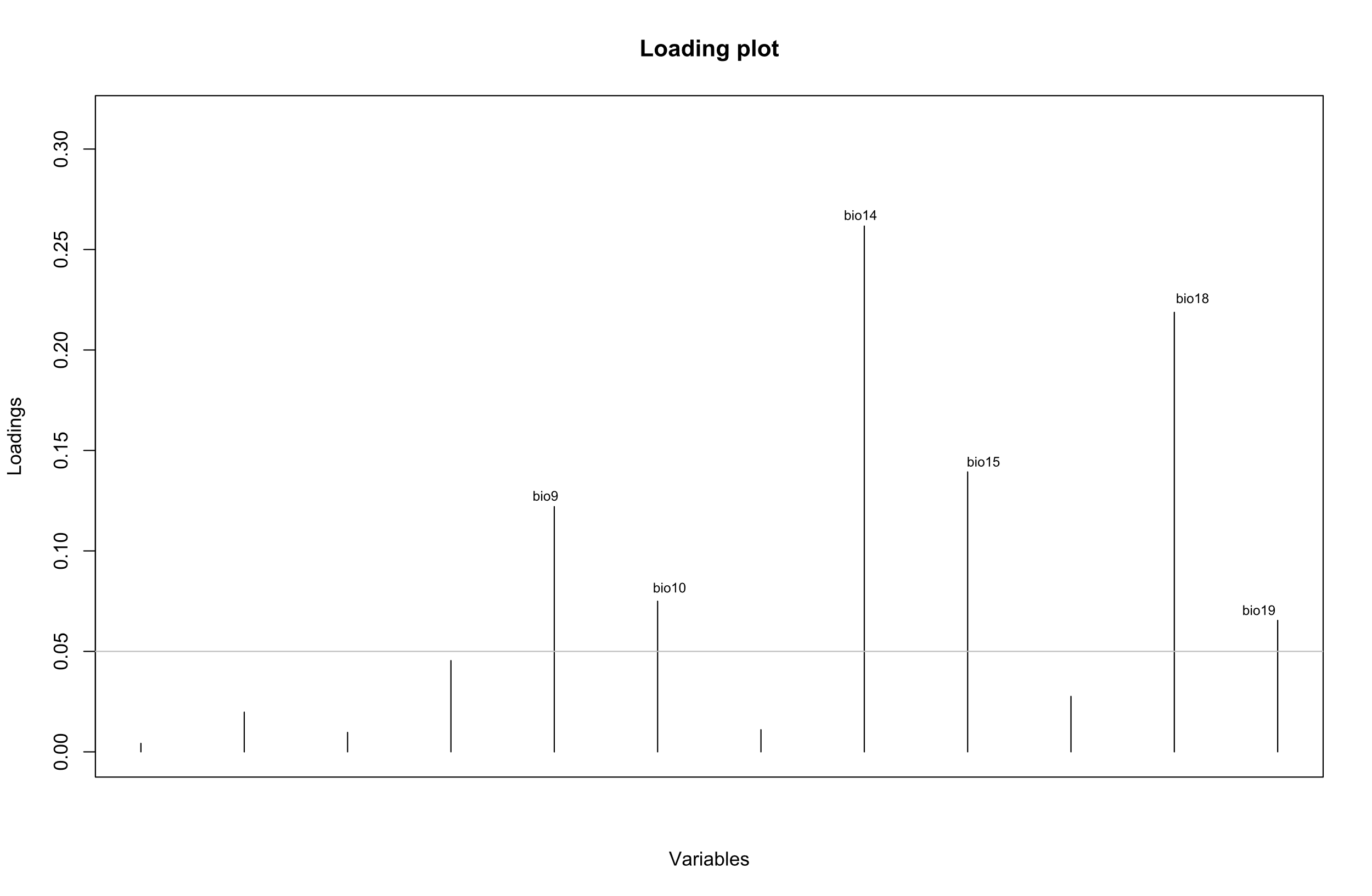


**Fig. S13:** Contribution of bioclimatic variables to the first two discriminant functions applying the threshold loading of 0.05. The most important bioclimatic variables were Bio14, Bio18, Bio15, Bio9, Bio10, and Bio19, respectively.


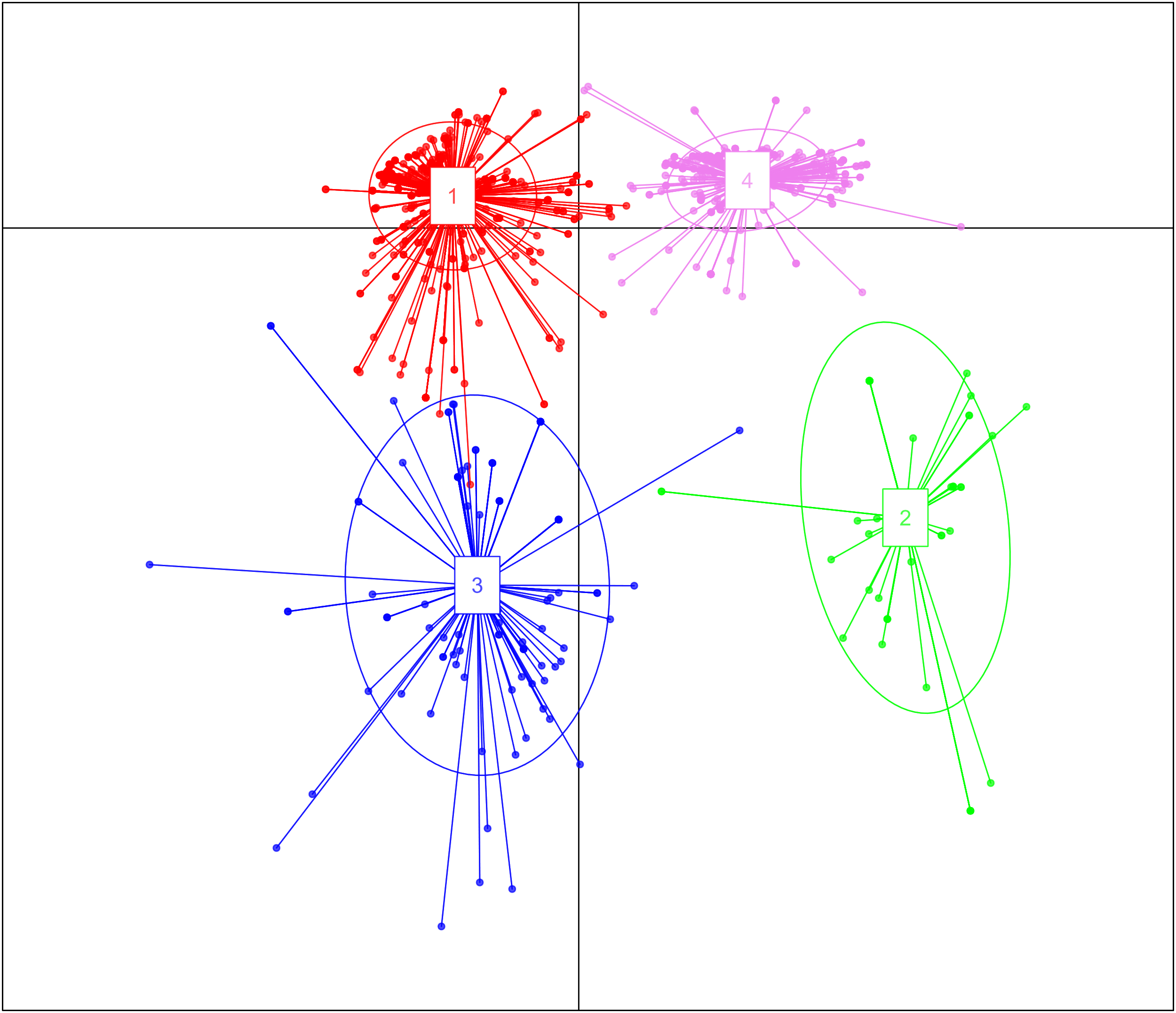


**Fig. S14:** DAPC biplot. Graphical representation for the first two discriminant functions. The plot indicates the separation of 1375 samples into four clusters.


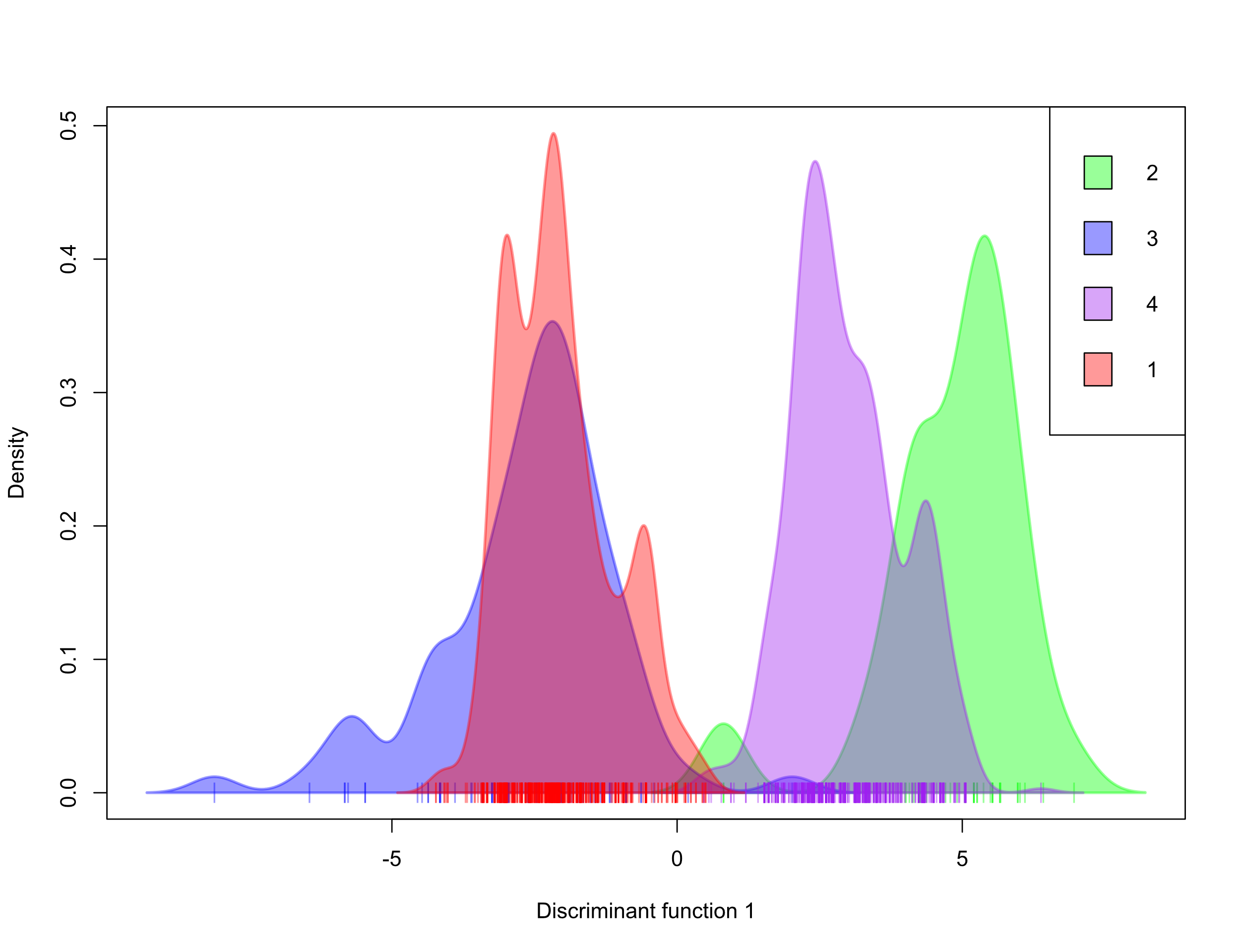


**Fig. S15:** Density plot of individuals along first discriminant function with different colors from the discriminant analysis of principal components (DAPC). The four clusters are shown.


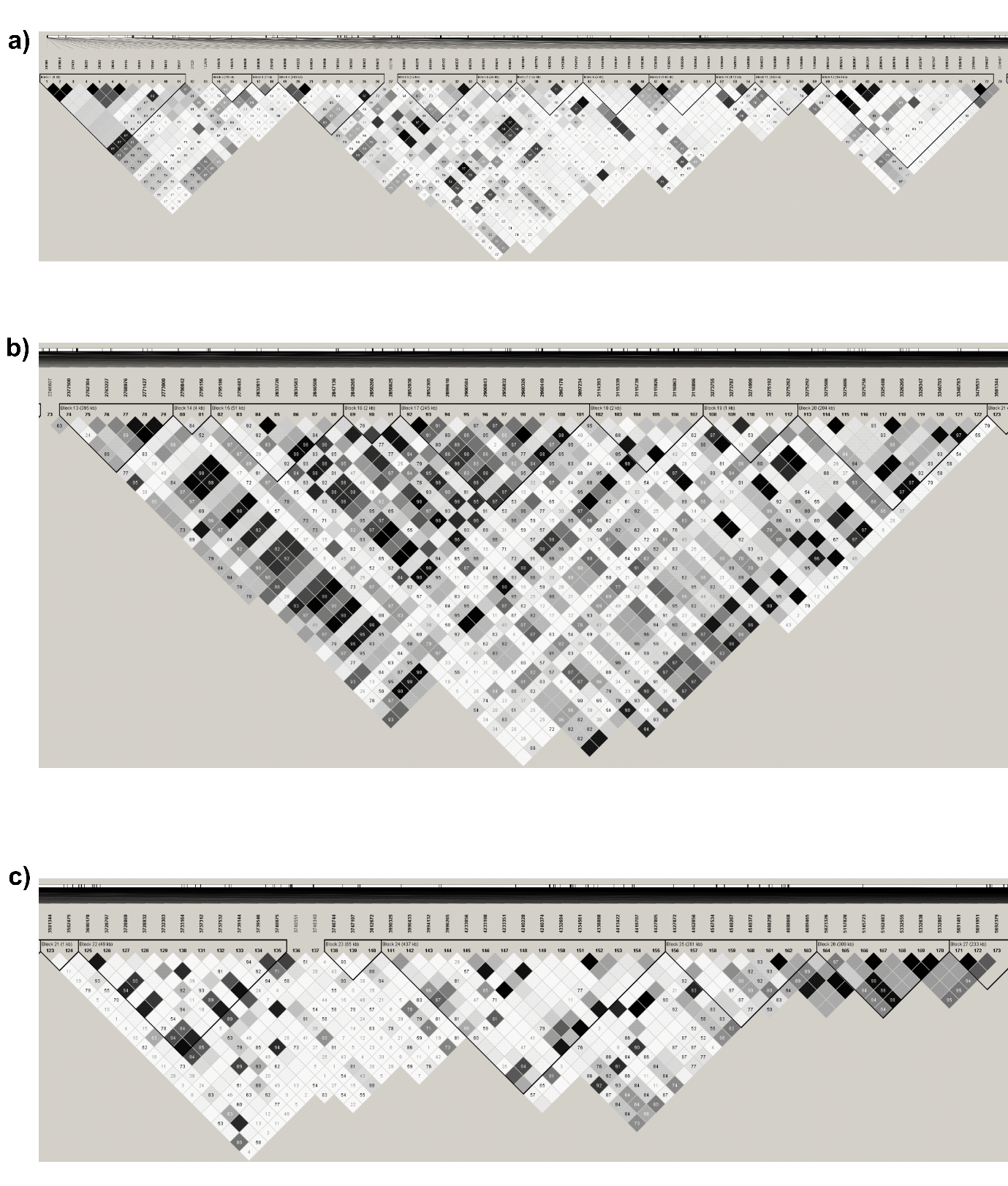


**Fig. S16**: Linkage disequilibrium (LD) patterns in the *PPD-H1* genomic region. a) LD blocks in the chromosomal region upstream of *PPD-H1*, b) LD blocks within the *PPD-H1* gene (29,123,785 to 29,127,889 bp) and c) LD blocks in the downstream region of the gene.

## **Supplementary References**

Casas A, Djemel A, Ciudad F, Yahiaoui S, Ponce L, Contreras-Moreira B, Gracia MP, Lasa J, Igartua E. 2011. HvFT1 (VrnH3) drives latitudinal adaptation in Spanish barleys. Theor Appl Genet. 122:1293-1304.https://doi.org/10.1007/s00122-011-1531-x

Herzig P, Maurer A, Draba V, Sharma R, Draicchio F, Bull H, Milne L, Thomas WTB, Flavell AJ, Pillen K. 2018. Contrasting genetic regulation of plant development in wild barley grown in two European environments revealed by nested association mapping. J Exp Bot. 69:1517-1531. <https://doi.org/10.1093/jxb/ery002>

Jombart T, Devillard S, Balloux F. 2010. Discriminant analysis of principal components: a new method for the analysis of genetically structured populations. BMC Genet 11:94. <https://doi.org/10.1186/1471-2156-11-94>

Le S, Josse J, Husson F. 2008. FactoMineR: an R package for multivariate analysis. *J Stat Softw* 25:1–18. https://doi.org/10.18637/jss.v025.i01

Maurer A, Draba V, Jiang Y, Schnaithmann F, Sharma R, Schumann E, Kilian B, Reif JC, Pillen K (2015) Modelling the genetic architecture of flowering time control in barley through nested association mapping. *BMC Genomics* 16 10.1186/s12864-015-1459-7. https://doi.org/10.18637/jss.v025.i01

Nei M. 1987. Molecular evolutionary genetics. New York (NY): Columbia University Press. ISBN 0-231-06320-2

Nitcher R, Distelfeld A, Tan C, Yan L, Dubcovsky J. 2013. Increased copy number at the *HvFT1* locus is associated with accelerated flowering time in barley. *Molecular Genetics Genomics* 288:261-275. https://doi.org/10.1007/s00438-013-0746-8

Payne, RW. 2009. GenStat. Wiley Interdisciplinary Reviews: Computational Statistics 1:255-258. https://doi.org/10.1002/wics.32

Untergasser A, Cutcutache I, Koressaar T, Ye J, Faircloth Brant C, Remm M, Rozen SG. 2012. Primer3--new capabilities and interfaces. *Nucleic Acids Res.* 40:e115. doi: 10.1093/nar/gks596

Wiegmann M, Maurer A, Pham A, March TJ, Al-Abdallat A, Thomas WTB, Bull HJ, Shahid M, Eglinton J, Baum M, Flavell AJ, Tester M, Pillen K. 2019. Barley yield formation under abiotic stress depends on the interplay between flowering time genes and environmental cues. *Sci Rep* 9:6397. https://doi.org/10.1038/s41598-019-42673-1

Zhang, Z, E Ersoz, C-Q Lai, et al. 2010. Mixed linear model approach adapted for genome-wide association studies. *Nature Genetics* 42:355-360. https://doi.org/10.1038/ng.546
